# Temperature, light and nitrate sensing coordinate Arabidopsis seed dormancy cycling, resulting in winter and summer annual phenotypes

**DOI:** 10.1111/tpj.12186

**Published:** 2013-04-17

**Authors:** Steven Footitt, Ziyue Huang, Heather A Clay, Andrew Mead, William E Finch-Savage

**Affiliations:** School of Life Sciences, Warwick UniversityWellesbourne Campus, Warwick, CV35 9EF, UK

**Keywords:** dormancy cycling, germination, *DELAY OF GERMINATION1*, environmental sensing, life cycle, nitrate, light, seedling emergence, hydrothermal time, *Arabidopsis thaliana*

## Abstract

Seeds use environmental cues to sense the seasons and their surroundings to initiate the life cycle of the plant. The dormancy cycling underlying this process is extensively described, but the molecular mechanism is largely unknown. To address this we selected a range of representative genes from published array experiments in the laboratory, and investigated their expression patterns in seeds of Arabidopsis ecotypes with contrasting life cycles over an annual dormancy cycle in the field. We show how mechanisms identified in the laboratory are coordinated in response to the soil environment to determine the dormancy cycles that result in winter and summer annual phenotypes. Our results are consistent with a seed-specific response to seasonal temperature patterns (temporal sensing) involving the gene *DELAY OF GERMINATION 1* (*DOG1*) that indicates the correct season, and concurrent temporally driven co-opted mechanisms that sense spatial signals, i.e. nitrate, via *CBL-INTERACTING PROTEIN KINASE 23* (*CIPK23*) phosphorylation of the *NITRATE TRANSPORTER 1* (*NRT1.1*), and light, via *PHYTOCHROME A* (*PHYA*). In both ecotypes studied, when all three genes have low expression there is enhanced *GIBBERELLIN 3 BETA-HYDROXYLASE 1* (*GA3ox1*) expression, exhumed seeds have the potential to germinate in the laboratory, and the initiation of seedling emergence occurs following soil disturbance (exposure to light) in the field. Unlike *DOG1*, the expression of *MOTHER of FLOWERING TIME* (*MFT*) has an opposite thermal response in seeds of the two ecotypes, indicating a role in determining their different dormancy cycling phenotypes.

## Introduction

Seeds can remain dormant in the surface layers of soil for many years until a time when the conditions are suitable for the resulting plant to thrive and reproduce (Finch-Savage and Leubner-Metzger, 2006; Footitt *et al*., 2011). To select this time, the depth of dormancy in the seed continually changes in response to a range of environmental signals that inform the seed about the seasons, its depth in the soil and the presence of competing plants. The precise response to these signals differs between species, and between ecotypes within species, through adaptation to the habitat and climate space they inhabit. The different seed dormancy cycles and resulting seasonal patterns of seedling emergence are well documented as a crucial component of the plants life cycle that contributes significantly to plant fitness (Donohue, 2002; Donohue *et al*., 2005; Huang *et al*., 2010; McNamara *et al*., 2011). However, the regulation of these phenological events at the molecular level has received little attention, despite the need to understand how these responses may adapt in the face of climate change.

Recent work investigating the molecular eco-physiology of dormancy cycling in field soils of the Arabidopsis ecotype Cape Verdi Isle (Cvi) revealed two forms of environmental sensing. One form relates to slow seasonal change (temporal sensing), with cycling from deep to shallow dormancy in order to select the time of year and climate space for emergence. This cycle is driven by the seasonal pattern of temperature, a finding that is consistent with earlier observations (Probert, 2000; Finch-Savage and Leubner-Metzger, 2006). The second form related to a rapid response to the suitability of local conditions for germination and establishment (spatial sensing). This work illustrates how molecular mechanisms identified as controlling dormancy in the laboratory could be seasonally coordinated in seeds buried in field soil to fulfill this process (Footitt *et al*., 2011).

The Arabidopsis ecotype Cvi exhibits the life cycle of a winter annual, by germinating in autumn and overwintering as a seedling rosette to produce dormant seeds that use the warmth of summer to relieve dormancy. By contrast, summer annuals shed their seeds in late summer, losing dormancy by exposure to low temperatures, so as to germinate in spring. Arabidopsis exhibits both patterns of annual behaviour (Baskin and Baskin, 1972; Donohue, 2002). Ratcliffe collected an ecotype (Bur) from the Burren in Ireland that flowered in September and behaved like a summer annual (Evans and Ratcliffe, 1972; Ratcliffe, 1976). Apart from these observations and its inclusion in a large-scale screening of germination conditions (Schmuths *et al*., 2006), no thorough investigation of its dormancy and germination behaviour has been reported. The two ecotypes Cvi and Bur with these contrasting life-cycle patterns naturally inhabit widely different environments in geographically distant regions (Bur, cool and damp; Cvi, warm and dry; see Figure S1), making them ideal for studying the differential adaptation of dormancy cycling and germination mechanisms.

We analysed dormancy regulation in Bur seeds buried in the soil, as depth of dormancy changed over an annual cycle, and compared it with that of Cvi seeds (Footitt *et al*., 2011). The results indicate that soil temperature drives seed-specific temporal sensing via the accumulation of *DELAY OF GERMINATION 1* (*DOG1*) protein to drive changes in germination potential. Unlike *DOG1*, expression of *MOTHER of FLOWERING TIME* (*MFT*) has an opposite thermal response in seeds of the two ecotypes, and may therefore have a role in their different dormancy cycling phenotypes. These seasonal responses are concurrent with changing sensitivity to nitrate and light (spatial sensing), which determine the actual time of germination. The response to nitrate appears to act via *CBL-INTERACTING PROTEIN KINASE 23* (*CIPK23*) phosphorylation/dephosphorylation of *NITRATE TRANSPORTER 1* (*NRT1.1*) and the response to light via *PHYTOCHROME A* (*PHYA*). This is consistent with the view that dormancy is an adaptive trait that arose evolutionarily late by co-opting pre-existing genetic pathways regulating other phase transitions (Bassel *et al*., 2011). In the field, when this temporal and spatial sensing overlapped with ambient environmental conditions, dormancy was removed and seeds progressed to germination completion and seedling emergence. Subtle adaptive differences in the patterns of temporal and spatial sensing can explain the winter and summer annual phenotypes of Cvi and Bur, respectively.

## Results

### Seasonal dormancy patterns in the Bur ecotype correspond to a summer annual phenotype

**Figure 1:**
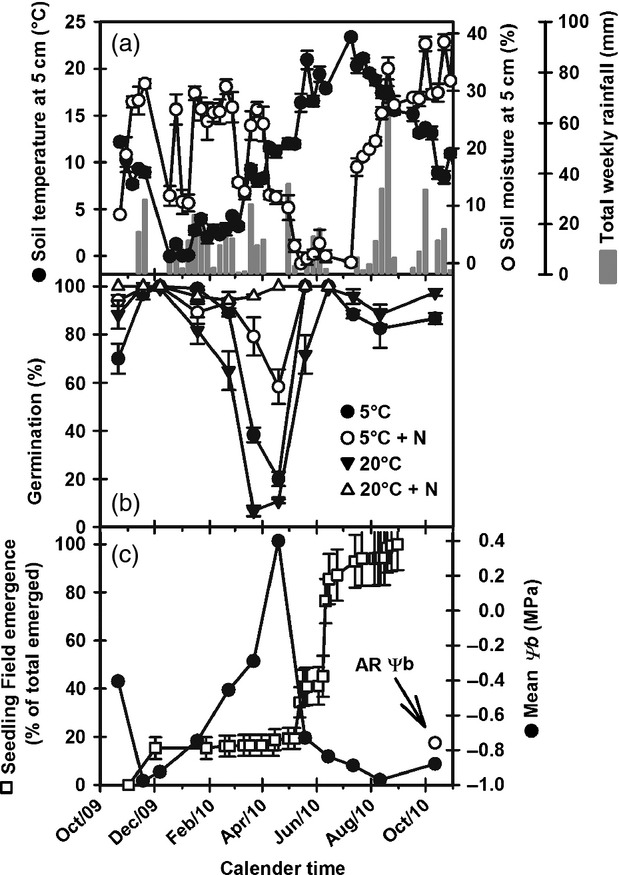
Seasonal changes in dormancy cycling. (a) Changes in soil temperature and moisture content measured at seed depth (5 cm), and weekly rainfall over 12 months from October 2009. (b) Changing thermodormancy in seeds recovered from the field. Following recovery, seeds where incubated in light at 5 and 20°C with and without 10 mm KNO_3_ (+N). (c) Mean seedling emergence following monthly soil disturbance (*n* = 3) is shown as the percentage of the total emerged. Mean base water potential (*Ψ*_b_) of seeds recovered from the field. Error bars indicate SEMs (*n* = 3).

To mimic the natural time of Bur seed dispersal (Ratcliffe, 1976), seeds were produced in late summer and buried in field plots in early October 2009. Soil temperature and moisture were recorded at seed depth to show the annual cycle of the soil environment, which seeds must sense to adjust their dormancy cycle (Figure 1a). Germination in the light at 5–25°C was high prior to burial, and initially increased in seeds exhumed following burial (Figure 1b and Figure S2). From December, thermodormancy then increased and germination decreased, first at higher temperatures, then at all temperatures. Dormancy reached a maximum in April, when germination in the light was lowest. Depth of dormancy then rapidly declined to a minimum over the next month. Sensitivity to nitrate was temperature dependent and declined at 5°C as dormancy increased, but remained level at 20°C at the relatively high concentration of 10 mm (Figure 1b). The nitrate response in Arabidopsis seeds is dose dependent (Alboresi *et al*., 2005; Finch-Savage *et al*., 2007), and thus lower levels of exposure under natural conditions would have a smaller effect on dormancy.

Bur seeds germinated in light following exhumation without the need for after-ripening; we therefore used changes in mean seed base water potential (*Ψ*_b_), calculated according to the hydrothermal time (HTT) model (Finch-Savage, 2004; Bradford, 2002), to indicate depth of dormancy. Before burial, seeds had a mean *Ψ*_b_ of −0.405 MPa. Following burial, the mean *Ψ*_b_ decreased to −0.976 MPa (Figure 1c) before increasing to a high point of 0.399 MPa in April when the seeds were most dormant (Figure 1b). *Ψ*_b_ then rapidly decreased as dormancy decreased. Seed viability was higher than 90% throughout the experiment, and seedling emergence occurred upon soil disturbance from early May to mid-June, demonstrating a summer annual habit (Figure 1c). The timing of seedling emergence was influenced by soil moisture, and therefore by rainfall (Figure 1a).

**Figure 2:**
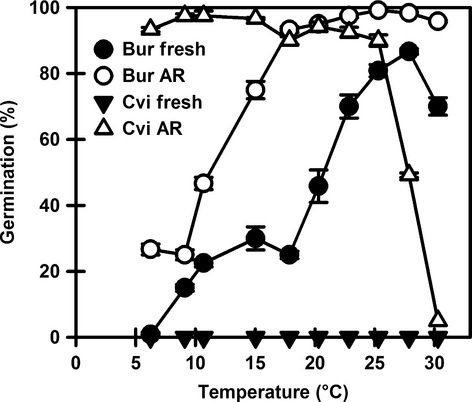
Thermal germination response of fresh and after-ripened Bur and Cvi seeds. Seeds of each ecotype where produced at the same time in a temperature-controlled glass house. Germination response was determined by incubating seeds on water in light on a thermogradient table with a linear temperature range of 5–31°C. The final germination percentage is shown for fresh and after-ripened seeds (225 days at 20°C following equilibration with 55% relative humidity).

Baskin and Baskin (1988, 1998) have shown that seeds from summer and winter annuals have characteristic responses to temperature that define life-cycle phenotypes. They show that seeds from summer annuals initially germinate at higher rather than lower temperatures, and then germinate at progressively lower temperatures as dormancy is lost. Seeds of winter annuals show the reverse of this pattern. We use these criteria to confirm our field observation above that Bur is a summer annual. At shedding, a greater percentage of Bur seeds germinated at higher temperatures than at low temperatures, and then progressively as dormancy was lost during after-ripening, seed germination at lower temperatures increased (Figure 2). For comparison, Cvi seeds were produced at the same time to avoid the influence of maternal conditions. In contrast to Bur, Cvi seeds exhibited the reverse response to temperature, i.e. were characteristic of a winter annual (Figure 2).

We sampled Bur seeds from the field experiment over the annual cycle to investigate the expression levels of key genes shown in laboratory studies to be involved in the regulation of seed dormancy (Finch-Savage and Leubner-Metzger, 2006; Finkelstein *et al*., 2008; Holdsworth *et al*., 2008; Footitt *et al*., 2011; Graeber *et al*., 2012). Gene family members were selected that exhibited distinct seed expression patterns in our previous laboratory-based microarray analyses of dormancy cycling (Cadman *et al*., 2006; Finch-Savage *et al*., 2007), as described in Appendix S1 and Footitt *et al*. (2011). Seeds were not exposed to light on exhumation, so the absolute requirement of Bur seeds for light to remove the final layer of dormancy was not fulfilled. Therefore, changes in gene expression represent changes in dormancy level in the soil seed bank.

### Gibberellic acid synthesis and signalling in Bur is consistent with a role in dormancy relief in late spring

**Figure 3:**
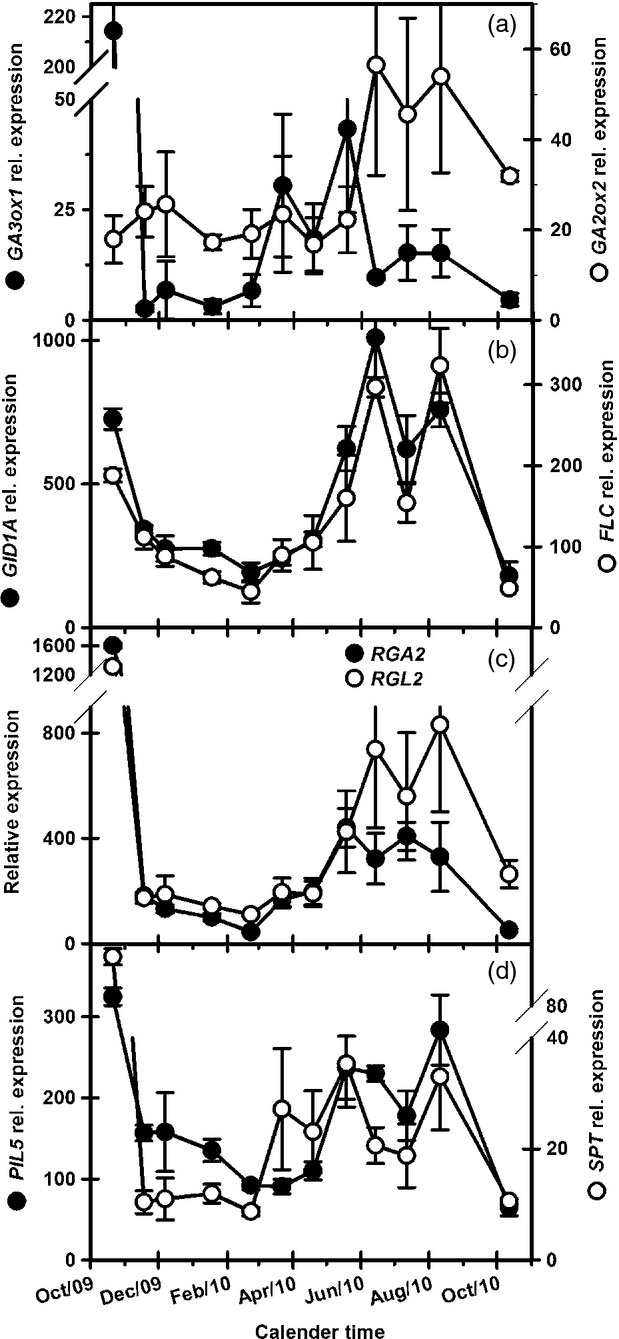
Gene expression in the GA biosynthesis and signalling pathway. (a) Expression of *GA3ox1* (GA biosynthesis) and *GA2ox2* (GA catabolism). (b) Expression of *GID1A* (GA receptor) and *FLC* (a flowering time regulator). (c) Expression of *RGA2* and *RGL2* (DELLAs – germination repressors). (d) Expression of *PIL5* and *SPT* (bHLH transcription factors of the PIF family – germination repressors). Error bars indicate SEMs (*n* = 3).

The expression of *GA2ox2* (gibberellic acid, GA, catabolism) was relatively stable over the winter, and then doubled in May after dormancy peaked in April (Figure 3a). In contrast, expression of *GA3ox1* (GA biosynthesis), decreased dramatically upon burial, only increasing as dormancy increased, and continued to increase up to 10-fold from its winter level as dormancy declined. *GA3ox1* expression then declined after May as *GA2ox2* increased, consistent with continual GA turnover, with synthesis dominating as dormancy declined.

The expression of *GID1A* (GA receptor) declined upon burial, with a small increase as dormancy increased in April/May, followed by a fourfold increase as dormancy declined (Figure 3b). *GID1A* expression then declined into autumn. Two DELLA genes (negative regulators of germination), *RGA2* and *RGL2*, both exhibited dramatic declines in expression upon burial, before following a pattern similar to *GID1A* (Figure 3c). Seeds remain dormant when exhumed in the dark; consistent with the high levels of DELLAs required to repress germination as dormancy declines. If the soil were disturbed, thereby exposing seeds to light, GA would increase dramatically (Cadman *et al*., 2006) to bind with GID1 and DELLAs to remove repression. Therefore, germination could occur rapidly when conditions fulfill those required for spatial sensing (Footitt *et al*., 2011).

**Figure 4:**
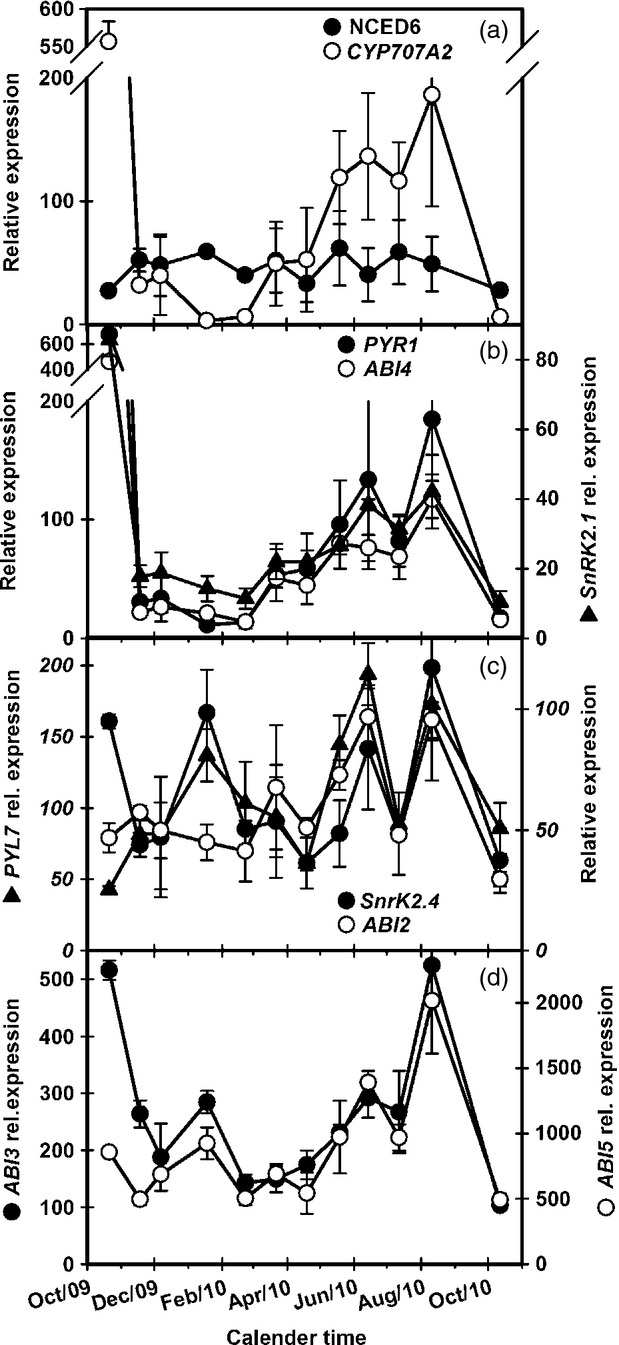
Gene expression in the ABA biosynthesis and signalling pathway. (a) Expression of *NCED6* (ABA biosynthesis) and *CYP707A2* (ABA catabolism). (b) Expression of *SnrK2.1*, an SNF1-related protein kinase subfamily member (positive regulator of ABA signalling), *PYR1* (ABA receptor) and ABI4 (control of energy use). (c) Expression of *PYL7* (ABA receptor), *SnrK2.4* (positive regulator of ABA signalling) and *ABI2* (repressor of ABA signalling). (d) Expression of *ABI3* (dormancy) and *ABI5* (ABRE-regulated transcription factor). Error bars indicate SEMs (*n* = 3).

The PHYTOCHROME INTERACTING FACTOR (PIF) family members, *PIL5* and *SPT*, show decreased expression upon burial. Expression then increased with dormancy before declining in the autumn (Figure 3d). *FLC* has a potential role in germination timing (Chiang *et al*., 2009), with an expression pattern identical to the GA receptor *GID1A* (Figure 3c). Expression of all GA signalling-associated genes, like ABA signalling genes (Figure 4), decreased in hot dry conditions before increasing with increased soil moisture following rainfall in July (Figure 1a).

### With the exception of NCED6, ABA synthesis and signalling genes, and those involved in nitrate and light sensing, show strong seasonal expression patterns in Bur

Expression of *NCED6* (ABA biosynthesis) changed little over the annual dormancy cycle (Figure 4a). In contrast, expression of *CYP707A2* (ABA catabolism) decreases dramatically upon burial, increasing again before and during the decline in dormancy. This is consistent with stable levels of endogenous ABA as dormancy increases, and decreasing levels as dormancy declines. This is in agreement with our observation that the increased depth of dormancy during cycling was not directly related to the endogenous ABA content of the seeds in Cvi (Footitt *et al*., 2011).

Upon burial, the ABA receptor gene *PYR1*, the SNF1-related protein kinase *SnRK2.1* and *ABI4* (negative regulator of germination) show dramatic declines in expression before increasing as dormancy declined in late spring (Figure 4b). The ABA receptor gene, *PYL7*, and the SNF1-related protein kinase, *SnRK2.4*, show increased expression as dormancy increased and, along with *ABI2*, increased again as dormancy declined in late spring (Figure 4c). The concurrent increase in the expression of ABA receptors and *SNRK2* genes (ABA signalling) with the negative regulator of ABA signalling ABI2 is counterintuitive. However, the decreasing depth of dormancy (temporal sensing, as shown by germination in light) with an increase in *ABI2* may need to be counterbalanced by a promotion of ABA signalling in the dark of the soil seed bank to prevent sensitivity to spatial signals. Of the ABA-induced transcription factors examined, *ABI4* expression increased as dormancy increased, and then remained elevated. Expression of *ABI3* and *ABI5* exhibited a double peak similar to that seen with *PYL7* and *SnRK2.4*.

**Figure 5:**
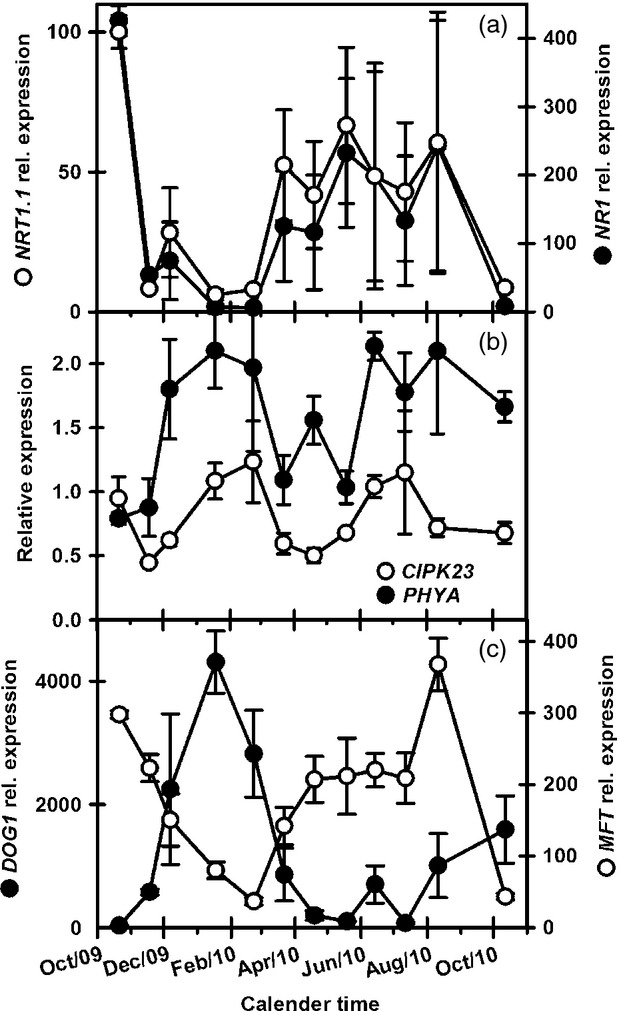
Gene expression related to temporal and spatial sensing. (a) Expression of *NRT1.1* (nitrate transporter/sensor) and *NR1* (nitrate reductase). (b) Expression of *CIPK23* (nitrate sensing) and *PHYA* (light sensing). (c) Expression of *DOG1* (dormancy) and *MFT* (ABA-induced germination repressor). Error bars indicate SEMs (*n* = 3).

Nitrate has a profound effect on seed dormancy (Hilhorst, 1990; Alboresi *et al*., 2005). Endogenous nitrate content of Arabidopsis seeds is negatively related to depth of dormancy, and enhances the effect of exogenous nitrate to relieve dormancy in the light (Alboresi *et al*., 2005; Matakiadis *et al*., 2009). *NRT1.1* (nitrate transporter) and *NRI* (nitrate reductase) had similar distinct expression profiles across the seasons (Figure 5a). CIPK23 is a regulator of NRT1.1 (Gojon *et al*., 2011), and PHYA) is linked to the regulation of dormancy via temperature (Heschel *et al*., 2008). The expression of both these genes in buried seeds had a similar overall pattern (Figure 5b); we argue below that they may be an integral part of the temporal regulation of dormancy cycling.

### Seasonal gene expression patterns are significantly associated with temperature in Bur and Cvi

In Bur, there were significant positive linear correlations (*P* < 0.05) between the expression levels of the majority of genes (Appendix S2); however, *SnrK2.4* shows few significant correlations, and strikingly neither *NCED6* (ABA synthesis) nor *GA3ox1* (GA synthesis) were significantly correlated with any other gene. In contrast, *CYP707A2* (ABA catabolism) and *GA2ox2* (GA catabolism) were significantly correlated with many genes (*P* < 0.05). The majority of these genes were also positively correlated with temperature and negatively correlated with soil moisture content (soil tends to be dry when temperature is high). The notable exception was *DOG1*, the expression of which was negatively correlated with soil temperature and therefore negatively related to most genes, although this relationship was only statistically significant with four genes, including *MFT*. *DOG1* is the gene at the locus with the strongest dormancy association in QTL analyses (Bentsink *et al*., 2006).

**Figure 6:**
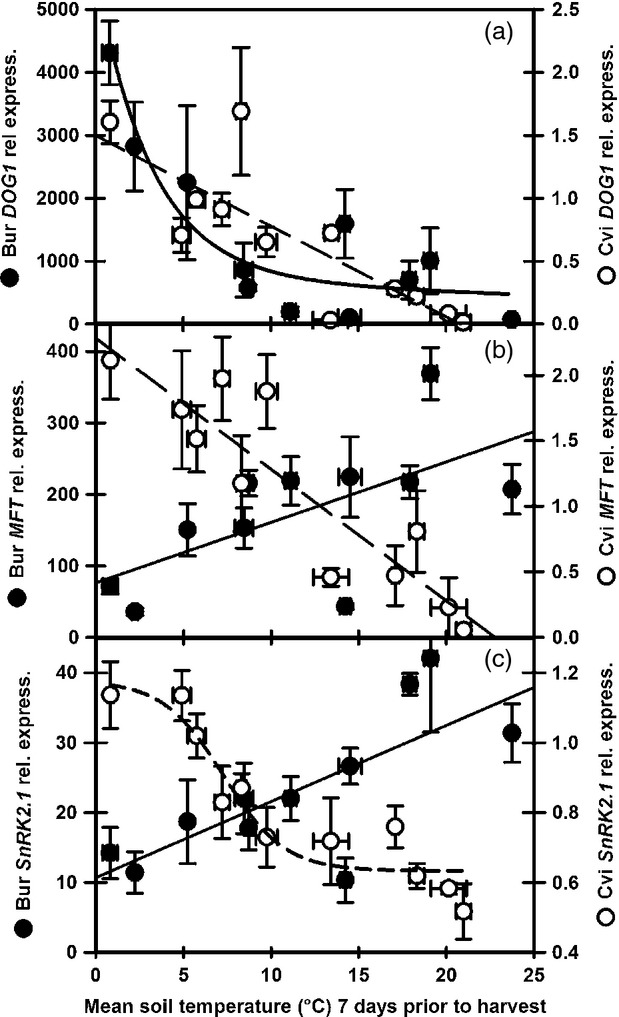
The impact of soil temperature on the expression of *DOG1* and *MFT* in Bur and Cvi. (a) The relationship between soil temperature and Bur and Cvi *DOG1* expression fits an exponential decay regression: Bur *f* = 4824.9840*exp(−0.313*x) + 775.2525*exp(−0.0207*x) (*R*^2^ = −0.8533); Cvi *f* = 0.8695*exp(−0.1023*x) + 0.9612*exp(−0.1023*x) (*R*^2^ = 0.6681). (b) The relationship between soil temperature and *MFT* expression fits a linear regression for Bur [*f* = 76.19 + 8.46(*x*); *R* = 0.63] and for Cvi [*f* = 2.28 − 0.10(*x*); *R* = 0.91]. (c) The relationship between soil temperature and *SnrK 2.1* expression fits a linear regression for Bur [*f* = 10.697 + 1.089(*x*); *R* = 0.75], and for Cvi fits a sigmoidal regression (*f* = 0.6331 + 0.5448/{1 + exp[−(*x* − 7.2894)/−1.7427]}; *R*^2^ = 0.8889). Cvi data were redrawn from Footitt *et al*. (2011).

When Bur and Cvi (Footitt *et al*., 2011) seeds exposed to the same general pattern of seasonal temperature are compared (Figure 1a), the resulting patterns of changing depth of dormancy differed dramatically. The gene expression patterns in the two ecotypes were compared to gain insight into the regulation of dormancy by temperature, revealing surprising similarities in *DOG1* expression, but contrasting expression patterns in *MFT* and *SnRK2.1*, with temperature (Figure 6). *DOG1* is significantly (*P* < 0.01 and *P* < 0.001, respectively) negatively correlated with soil temperature in both Bur and Cvi, whereas, *MFT* and *SnRK2.1* are significantly (*P* < 0.05 and *P* < 0.01, respectively) positively correlated with soil temperature in Bur, but are negatively correlated in Cvi (both *P* < 0.001; Appendices S2 and S3; Footitt *et al*., 2011).

**Figure 7:**
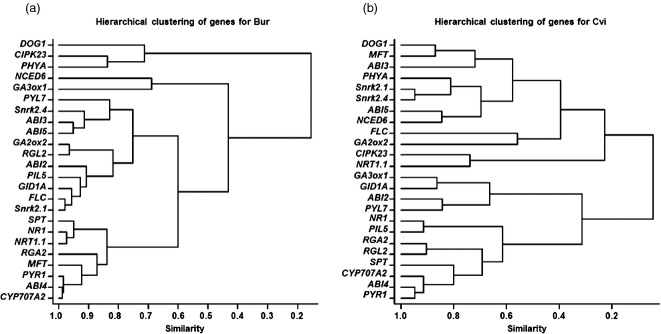
Dendrograms from hierarchical cluster analysis of gene expression patterns in Bur and Cvi ecotypes during the annual dormancy cycle: (a) Bur; (b) Cvi. Clusters group genes together that have similar expression patterns (i.e. close to one). The analysis is described in Appendix S1.

We carried out cluster analysis to look for similarities in gene expression patterns within ecotypes, and then applied the Mantel test to look for associations between the ecotypes. Overall, there was a significant association (*P* < 0.001) between the expression patterns of individual genes. In Bur, *DOG1*, *CIPK23* and *PHYA* were clustered together with the greatest level of similarity, and were separate from the other genes in the dendogram (Figure 7). These genes were clustered more loosely in Cvi, but in both ecotypes these genes were separated from six genes clustered in Bur and Cvi (*CYP707A2*, *PYR1*, *ABI4*, *RGA2*, *SPT* and *NRI*). These clusters were confirmed using principal component analysis (PCA; Figure S3). Further confirmation of differences between ecotypes was shown using Procrustes rotation to compare the PCA configurations.

In a further analysis the across-gene profiles in the PCA were then regressed with the seasonal patterns of temperature and the pattern of change in depth of dormancy (Bur using *Ψ*_b_ and Cvi using AR50). We found that 76 and 47% of the variation in gene expression pattern in the first principal component could be explained by the temperature profile in Cvi and Bur, respectively. In deeply dormant Cvi 53% of the variation in dormancy was also associated with the across-gene expression profiles, clearly indicating the importance of the genes selected in regulating dormancy. However, in the less dormant Bur only 0.7% of the variation in dormancy could be explained by the expression pattern of these same genes.

## Discussion

### The phenology of dormancy in the Bur and Cvi ecotypes is consistent with summer and winter annual phenotypes, respectively

**Figure 8:**
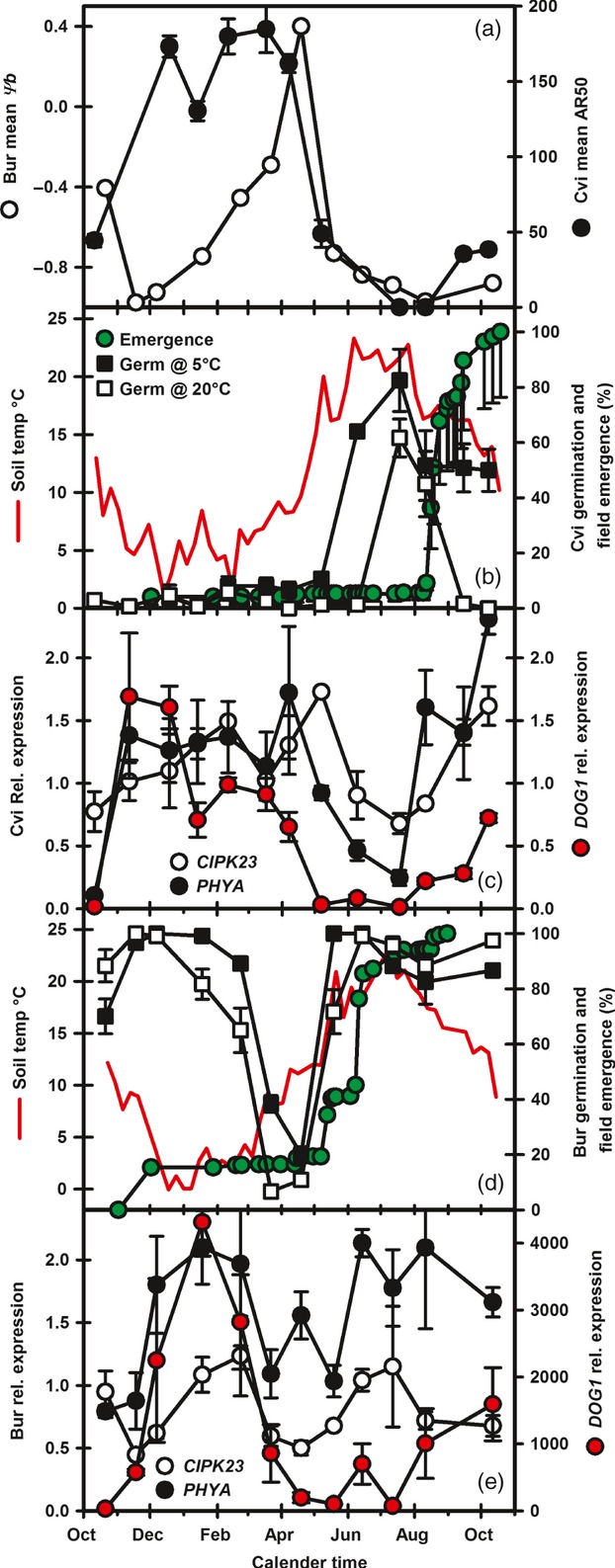
Seasonal coordination of germination and seedling emergence through temperature, light and nitrate sensing. (a) Depth of dormancy in Bur [base water potential (*Ψ*_b_)] and Cvi [time to 50% after-ripening (AR50)] (Footitt *et al*., 2011). (b) Changing thermodormancy and seedling emergence of Cvi in relation to the annual soil temperature cycle (2007–2008) (Footitt *et al*., 2011). (c) Expression of *DOG1* (temperature sensing; Footitt *et al*., 2011), *PHYA* (light sensing) and *CIPK23* (nitrate sensing) in Cvi. (d) Changing thermodormancy and seedling emergence of Bur in relation to the annual soil temperature cycle (2009–2010). (e) Expression of *DOG1* (temperature sensing), *PHYA* (light sensing) and *CIPK23* (nitrate sensing) in Bur.

In the laboratory, seeds of Bur and Cvi produced under the same maternal conditions exhibited germination responses to temperature that were characteristic of summer and winter annuals, respectively (Figure 2; Baskin and Baskin, 1988, 1998). We compare the seasonal dormancy patterns of these contrasting ecotypes in Figure 8(a). Both ecotypes still required light, and thus seeds were dormant throughout the annual cycle in the soil. At burial, Bur seeds showed minimal dormancy, and after 1 month complete germination was seen in the presence of light at all temperatures tested; however, dormancy then increased over winter to April. Dormancy in Cvi also increased over this period, even though dormancy was much deeper at burial. Strikingly, dormancy then declined rapidly in both ecotypes during May, and then continued to decline more slowly. Thus temporal sensing is similar in both ecotypes. Nevertheless, in field plots where the soil was disturbed regularly, exposing seed to light, Cvi seedlings began emerging in August (Figure 8b; Footitt *et al*., 2011), whereas Bur seedlings emerged 2 months earlier (Figure 8d), consistent with winter and summer annual phenotypes, respectively.

Closer inspection of Figure 8(b,d) shows that thermodormancy changes more slowly in Cvi than Bur, even though overall dormancy loss is coincident (Figure 8a). Germination potential at 5°C in light increased over a similar period in April in both ecotypes. In contrast, the potential to germinate at 20°C in light in Bur follows rapidly, but in Cvi germination at 20°C is delayed until July. Following the loss of dormancy in Bur by November, secondary dormancy appears to be induced, with thermodormancy apparently reverting to that seen in the WA ecotype Cvi, and more pronounced at higher temperatures (compare Figures 1, 2, and 8). This has implications for the adaptation of ecotypes over generations (Pico, 2012).

The combination of the slower opening of the temperature window for germination coupled with increasing ambient soil temperature in spring results in later germination and seedling emergence of Cvi compared with Bur following soil disturbance and exposure to light. We interpret these results below, by considering dormancy as a continuum (Cadman *et al*., 2006; Finch-Savage and Leubner-Metzger, 2006; Finch-Savage *et al*., 2007; Footitt *et al*., 2011; Finch-Savage and Footitt, 2012), without separate dormancy relief and germination stimulation processes (*sensu* Vleeshouwers *et al*., 1995; Thompson and Ooi, 2010), an approach that is justified elsewhere (Finch-Savage and Footitt, 2012).

### Expression of hormone balance and signalling genes during dormancy cycling in buried seeds is consistent with their functions in dormancy regulation proposed in laboratory experiments

In Cvi the annual dormancy pattern in the field is explained by the expression patterns of hormone biosynthesis and signalling genes (Footitt *et al*., 2011). Slow seasonal changes in depth of dormancy (temporal sensing) were linked with ABA signalling coupled to DELLA repression during low dormancy (spatial sensing). In the less dormant Bur, gene expression patterns showed little evidence of the slow seasonal response and deep dormancy, characterized by the increased *NCED* and *SNRK* expression seen in the deeply dormant Cvi (Footitt *et al*., 2011). In contrast, the seasonal pattern in Bur was dominated by long periods of low dormancy. In practice, low temperatures in autumn would initially prevent germination following shedding, as dormancy increased. As dormancy subsequently declined, *GA3ox1* expression peaked and *GA2ox2* remained the same (Figure 4a), consistent with an increase in GA and the seedling emergence observed following disturbance in May/June (Figure 8d). *GA3ox1* expression then dramatically decreased as *GA2ox2* expression and *DELLA* expression increased (Figure 4c), consistent with inhibiting germination, a control that is rapidly removed by increased GA resulting from exposure to light by soil disturbance. Thus hormone synthesis, catabolism and signalling gene expression is consistent with their proposed functions in the downstream regulation of depth of dormancy in buried seeds.

### DOG1 expression and dormancy have seasonal patterns determined by temperature (temporal sensing)

Gene expression patterns in the soil relate to the changing depth of dormancy resulting from environmental sensing. *DOG1* is the only gene studied in which expression is significantly (*P* < 0.01) and negatively related to the annual pattern of soil temperature in both ecotypes (Appendix S2). *DOG1* expression increases upon burial in Cvi (Footitt *et al*., 2011), but is initially delayed in Bur as dormancy is lost. As dormancy then increases (secondary dormancy), *DOG1* expression also increases. *DOG1* expression therefore increases in a similar fashion with depth of dormancy (Figure 8a,c,e) in both ecotypes, but dormancy does not decline immediately when *DOG1* expression declines.

In Cvi, although ABA was required, the absolute level of ABA did not set the depth of dormancy, and *DOG1* expression appeared to be the dominant factor influencing ABA signalling (Footitt *et al*., 2011). This is supported by observations during seed maturation (Chiang *et al*., 2011; Kendall *et al*., 2011), where DOG1 and ABA function in largely independent pathways, with DOG1 indirectly enhancing ABA synthesis (Nakabayashi *et al*., 2012). Thus DOG1 does not regulate dormancy primarily via changes in hormone levels, and the environment independently influences ABA and DOG1 (Footitt *et al*., 2011; Nakabayashi *et al*., 2012).

We suggest that following shedding, DOG1 in the presence of ABA responds to temperature, becoming a seed dormancy-specific thermal sensing mechanism driving the slow seasonal response (temporal sensing; Footitt *et al*., 2011). DOG1 protein accumulates during seed maturation, remaining stable during storage, and its modification during after-ripening acts as a timer for seed dormancy release (Nakabayashi *et al*., 2012). Here, in both Bur and Cvi, dormancy increases as *DOG1* expression increases, and declines after *DOG1* expression declines. The difference between the ecotypes is that Cvi is more deeply dormant at maturation and before burial than Bur, probably having accumulated more DOG1. Small genetic differences in seed DOG1 protein levels at maturity can correlate positively with depth of dormancy (Nakabayashi *et al*., 2012). In the present study we have used ecotypes that exhibit winter and summer annual characteristics when produced under the same conditions (Figure 2), and therefore DOG1 protein levels would result from a genetic difference. However, *DOG1* expression is also altered by maturation environment (Kendall *et al*., 2011). This suggests the intriguing possibility that by altering DOG1 protein levels to set different dormancy levels at maturity, maturation conditions alter subsequent dormancy, and therefore life-cycle behaviour, via DOG1 accumulation and loss. Indeed, life-cycle phenotypes (summer and winter annuals) can occur in the same population, with proportions changing systematically with temperature along an altitude gradient (Pico, 2012). There is also evidence that the low maturation temperatures that deepen dormancy may alter the behaviour from winter to summer annuals (Kendall *et al*., 2011). Taken together, this suggests that DOG1 may have a central role in determining not just dormancy level, but also life-cycle phenotype.

In Cvi, the expression profiles of *DOG1*, *MFT* and *SnrK2.1* are negatively related with temperature (Footitt *et al*., 2011), whereas in Bur the relationship is positive for *MFT* and *SnrK2.1* (Figure 6). This contrasting relationship with temperature may result from natural variation driven by adaptation of these ecotypes to widely different habitats, and is potentially significant when seed response to temperature also differs between the ecotypes (Figure 2). However, the role of MFT is not clear. MFT is a proposed ABA-induced negative regulator of ABA signalling that promotes embryo growth in the germinating seeds of Arabidopsis (Xi *et al*., 2010). In this work the seeds were after-ripened and also stratified, and so were non-dormant. In contrast, a study in *Triticum* spp. (wheat) showed expression of MFT increased after physiological maturity in dormant seeds produced at the lower temperatures that enhanced dormancy (Nakamura *et al*., 2011). They considered MFT as a candidate gene for seed dormancy regulation, and showed that transient overexpression of MFT in immature wheat embryos enhanced dormancy and prevented germination. Thus the two studies indicate a different role for MFT, but it is not clear whether the difference results from species differences or from the different states (dormant or non-dormant) that were studied. If the latter, then the different patterns of *MFT* expression shown here may be a consequence of, or part of, the natural variation in depth of dormancy in the two ecotypes. In the weakly dormant Bur, *MFT* expression rises as *DOG1* expression declines, whereas, in the deeply dormant Cvi *MFT* expression peaks after that of *DOG1* and remains high as *DOG1* declines. This is consistent with *MFT* in an antagonistic role to *DOG1*, and arguably consistent with MFT as the convergence point of ABA and GA signalling pathways (Xi *et al*., 2010). MFT could therefore influence sensitivity to spatial signals in response to temporal patterns in the dark of the soil seed bank. In support of this hypothesis, like *DOG1*, Nakamura *et al*. (2011) report that *MFT* expression is regulated in response to temperature, and seems to transmit temperature signals to a downstream temperature-signalling cascade to regulate depth of seed dormancy.

### Germination and seedling emergence are initiated by a temporal shift in sensitivity to nitrate and light (spatial sensing), potentially via CIPK23 and PHYA

When seeds gain the potential for germination through temporal sensing, germination can occur when the temperature window is coincident with ambient temperature. However, mechanisms that can respond to other spatial signals have to be in place and satisfied before dormancy is fully removed and seeds can complete germination. We consider two spatial signals here, nitrate and light: the latter is an absolute requirement in both ecotypes, whereas the former is only absolutely required by Bur, and then only for a short period in April.

#### Nitrate

Seed dormancy can be released by nitrate in Arabidopsis, but it is not clear whether nitrate acts on seed germination itself or through the production of N-related signals (Alboresi *et al*., 2005); however, nitrate accelerates the decrease in ABA prior to the completion of germination (Ali-Rachedi *et al*., 2004) via the induction of the catabolic ABA gene *CYP707A2* (Matakiadis *et al*., 2009). Despite these clear laboratory results, a review of the literature on the involvement of nitrate in dormancy cycling (Appendix S4) indicates that endogenous nitrate content has little ecological significance (Bouwmeester *et al*., 1994), and that even if it changes with the seasons soil nitrate content has little impact on dormancy cycling. It was suggested that temperature results in reversible changes in sensitivity to nitrate (and light, as seen for Bur; Figure 1b) at the level of receptors, and that control via the availability of receptors is likely (Bouwmeester and Karssen, 1993; Derkx and Karssen, 1993; Bouwmeester *et al*., 1994). This is consistent with the earlier conclusions of Hilhorst (1990) in a laboratory study of secondary dormancy.

Alboresi *et al*. (2005) suggest that the nitrate receptor hypothesized by Hilhorst (1990) could be NRT1.1, a view supported by reviewing current literature (Appendix S4) and our earlier laboratory dormancy cycling array data (Cadman *et al*., 2006; Appendix S5). NRT1.1 is a dual-affinity nitrate transporter, with a high- or low-affinity function depending on the phosphorylation status of threonine-101 (T101; Ho *et al*., 2009), and is considered to be a nutrient transceptor (duel nutrient transport/signalling function; Gojon *et al*., 2011). In low-nitrate conditions T101 of NRT1.1 is phosphorylated by CIPK23, transforming it to a high-affinity transporter, whereas, in high-nitrate conditions CIPK23 is not required. Thus in seeds, as high nitrate releases dormancy, we speculate that low *CIPK23* expression equates to low dormancy. In agreement with this hypothesis, laboratory dormancy cycling transcriptomes in Cvi show significantly lower *CIPK23* in low-dormancy and non-dormant states, but enhanced expression in the deeply primary dormant (PD30) and secondary dormant (SD1, SD2) states, with low nitrate sensitivity (Cadman *et al*., 2006; Appendix S5). There was no significant pattern in other downstream components of the nitrate signalling pathway, i.e. *CIPK8* and *ANR1* (Appendix S5). These data suggest that Cvi seeds in the laboratory held in the dormant state may uncouple the signalling and transport function of NRT1.1 to reduce sensitivity to nitrate and deepen dormancy.

Intriguingly, a comparison of changing depth of dormancy and the expression pattern of *CIPK23* shows clear correspondence in both Bur and Cvi (Figure 8c,e). Higher expression coincides with increasing dormancy and lower expression coincides with lower dormancy and seedling emergence in the field. There is some delay, as emergence results from subsequent seedling growth and is subject to the water available in the soil. In Cvi there is a single dip in expression in late July, coincident with field emergence. In Bur, expression levels undulate, but there are two main flushes of seedling emergence that follow the periods of low *CIPK23* expression, coincident with low *DOG1* and *PHYA* expression (Figure 8d,e). Multiple flushes are possible in Bur since, in contrast to Cvi, dormancy is low throughout much of the year. Thus, the seed may be mimicking a high nitrate situation (relieves dormancy) to release dormancy via the same phosphorylation/dephosphorylation switch to elicit a downstream dormancy-related signalling cascade. This is possible because nitrate transport activity is not required for the sensing function (Ho *et al*., 2009). It is important to point out that endogenous nitrate content is positively related to lower dormancy, and enhances the concentration-dependent effect of exogenous nitrate in Arabidopsis (Alboresi *et al*., 2005; Matakiadis *et al*., 2009); however, this does not explain the greater response of Bur seeds to nitrate, as nitrate levels were greater in Cvi (439 ± 20.8 mg kg dry weightt^−1^) than in Bur (208 ± 14.6 mg kg dry weightt^−1^) seeds used in these experiments.

#### Light

Phytochomes are among the most important sensors in plants that respond not only to light, but also to multiple seasonal cues (Heschel *et al*., 2008). In the field, mutant studies show phytochromes to be important in the control of seasonal germination timing, which is strongly influenced by seed maturation conditions (Donohue *et al*., 2012). Our microarray data show that during dormancy cycling only the phytochromes *PHYA* and *PHYD* have strong dormancy-associated expression patterns in dark-imbibed seeds (Appendix S5; Cadman *et al*., 2006; Finch-Savage *et al*., 2007). These data show *PHYA* expression is highest when seeds are deeply dormant (relative expression in non-dormant seeds LIG = 593; in dormant seeds imbibed fpr 24 h, PD24 = 773; in the deeply dormant seeds resulting from prolonged warm temperatures, PD30 = 2887; and resulting from prolonged low temperatures, SD2 = 2996). Partially after-ripened seeds exposed to low temperatures or nitrate in the dark also have high *PHYA* expression (PDC = 2810; PDN = 2292), whereas those exposed to light have low expression (PDL = 687), even when in the presence of nitrate (PDLN = 718); dormancy remains in all four treatments and seeds will not complete germination. High *PHYA* expression therefore appears linked to prolonged exposure in the dark and to low temperature and nitrate, conditions that exist during dormancy cycling in the field.

Interestingly, with short imbibition periods in the laboratory, the function of PHYA in seeds has been shown to act via the very low fluence response (VLFR), which is saturated at the levels of active phytochrome (Pfr), and which are lower than those produced with FR filters, and is consequently not red(R)/far red(FR) reversible (Botto *et al*., 1996; Shinomura *et al*., 1996). The VLFR may be induced by light flashes of tenths of a second of sunlight exposure (Scopel *et al*., 1991; Botto *et al*., 1998), thereby promoting germination during disturbance of the soil seed bank, for example during tilling (Botto *et al*., 2000). PHYA also operates to inhibit germination via the high irradiance response (HIR) produced when vegetation cover reduces the R/FR ratio of incident light (Batlla *et al*., 2000; Shichijo *et al*., 2001). However, studies with Arabidopsis mutants show that PHYA contributes to cold-induced dormancy and represses germination in white light in seeds matured at low temperature in both low and neutral R/FR conditions (Donohue *et al*., 2008; Heschel *et al*., 2008; Dechaine *et al*., 2009). PHYD was redundant with regard to germination inhibition or the maintenance of dormancy. That PHYA inhibits germination under both R/FR conditions indicates that maturation temperature is dominant.

Based on the information above we focused on the expression of *PHYA* in seed recovered from the soil seed bank. In the cold and dark of the soil seed bank *PHYA* expression increased, consistent with our microarray data (Appendix S5), and with the observation that PHYA protein accumulates rapidly in dark-imbibed seeds but not in light, and in seedlings 85% of phytochrome protein was PHYA in dark-grown seedlings compared with only 5% in light-grown seedlings (Sharrock and Clack, 2002).

Increased *PHYA* expression in seeds exhumed from the soil seed bank (Figures 4b and 7) and prolonged dark exposure in the laboratory (Appendix S5; Cadman *et al*., 2006; Finch-Savage *et al*., is negatively correlated with *Ga3ox1* expression (Bur, *r* = −0.845, *P* > 0.05 from December onwards; Cvi, *r* = −0.700, *P* > 0.05). This behaviour is consistent with reports that *PHYA* overexpression results in reduced *Ga3ox1* expression and reduced GA levels (Jordan *et al*., 1995; Foo *et al*., 2006). Germination of Cvi seeds recovered from the field were GA insensitive when *PHYA* expression was high (Footitt *et al*., 2011; Figure 8c). Taken together, this suggests a role for *PHYA* in inhibiting the germination of seeds exposed to light by soil disturbance in the field when they are outside the seasonally determined emergence window (Figure 8b,c,d,e). This interpretation is further supported by the PHYA inhibition of germination in continuous and intermittent red light (Appenroth *et al*., 2006).

In both ecotypes *PHYA* expression is the inverse of PIF genes, *PIL5* and *SPT* (*PHYA* versus *SPT* in Cvi, *r* = −0.787, *P* > 0.01; Appendix S3), and DELLA gene expression. *PIL5* and *SPT* repress *Ga3ox1*, with phytochromes repressing *PIL5* and *SPT*, and with cold also repressing *SPT* (Penfield *et al*., 2005; Oh *et al*., 2006); however, *SPT* and *GA3ox1* are positively correlated in both ecotypes (Appendix S3). In winter and spring when *PIF* and *DELLA* gene expression is low, PHYA may be able to repress GA3ox1 and potentially other aspects of GA signalling by an independent pathway. Thus seeds in the soil seed bank are protected from inappropriate germination following soil disturbance and exposure to light in an unfavourable season.

### Coordination of temperature, light and nitrate sensing is required to determine the timing of germination in the field

We have used natural variation between contrasting Arabidopsis ecotypes to investigate the coordination of dormancy-regulating mechanisms, characterized in the laboratory, to regulate dormancy cycling in field soils. We show that the expression patterns of *DOG1*, *PHYA* and *CIPK23* cluster in both Arabidopsis ecotypes (Figure 7), and that all three appear to act as temporal sensors and negatively regulate germination. When all three genes have low expression (Figure 8c,e) there is enhanced *GA3ox1* expression (Figure 4a; Footitt *et al*., 2011), and seeds have the potential to germinate (Figure 8b,d). From this data we hypothesize that there is a well-conserved seasonal (temporal) seed-specific sensing of temperature via DOG1 that is linked to other temporal responses that alter sensitivity to spatial signals through co-opted sensing mechanisms: i.e. nitrate via CIPK23 phosphorylation of NRT1.1 and light via PHYA. Seed dormancy is then altered via downstream hormone signalling. In the contrasting ecotypes shown, the initiation of seedling emergence following disturbance (exposure to light) in the field is coincident with the lowest levels of *DOG1*, *CIPK23* and *PHYA* expression (Figure 8). Thus when the spatial signals of appropriate temperature, nitrate and light are satisfied at this time, completion of germination results and seedlings emerge, subject to ambient soil moisture and temperature conditions (Figure 8b,d). Different dormancy levels set in response to environmental conditions during maturity are likely to influence the pattern of this dormancy cycle.

The patterns of gene expression in response to environmental signals are subtly different in the two ecotypes, providing insight into how adaptation to local conditions can generate winter and summer annual phenotype behaviour. Our hypothesis is consistent with physiological and ecological views on the timing of dormancy loss in response to overlapping environmental signals for seasonal and gap sensing that establish the resulting plant in the most appropriate climate space, habitat, and time for growth and reproduction.

## Experimental procedures

Seeds were produced (May–July 2009) in a temperature-controlled glasshouse. Mature seeds were harvested in July by hand threshing and equilibrated at 55% relative humidity at 20°C for 7 days to produce an equilibrium moisture content of 6–10% on a dry-weight basis. Seeds were stored at −80°C in sealed tubes. Seeds were dispersed in soda lime Ballotini balls, then placed in nylon mesh bags and buried in the field at a depth of 5 cm, before being recovered in the dark and processed as described previously (Footitt *et al*., 2011; details of seed burial, seedling emergence, germination tests, base water potential determination and subsequent analysis are described in Appendix S1).

Gene expression in seed RNA was analysed using the Nanostring ncounter gene expression system (Geiss *et al*. 2008). Details of Nanostring probes are presented in Table S1. The expression of additional genes was determined by quantitative PCR performed in triplicate on each of three independent biological samples. Gene expression levels were determined using a cDNA dilution series of the primer pairs of each gene of interest, with normalization against the housekeeping gene *At4 g34270* (*Tip41-like*; further details are given in Appendix S1).

## SUPPORTING INFORMATION

**Figure S1.** Annual weather patterns for the Burren, Ireland and Cape Verde Islands.

**Figure S2.** Changing thermodormancy in seeds recovered from the field.

**Figure S3.** Principal component analysis of gene expression profiles over time in (a) Bur and (b) Cvi.

**Table S1.** Probe sets for genes of interest used for Nanostring analysis.

**Appendix S1.** Methods.

**Appendix S2.** Linear correlation coefficients for comparisons between environmental, physiological and molecular parameters for buried Bur seeds recovered from the field.

**Appendix S3.** Comparison of significant correlations between Bur and Cvi ecotypes.

**Appendix S4.** A review of the influence of nitrate on dormancy cycling.

**Appendix S5.** Expression heat maps of *TIP41-like* reference gene and genes involved in light and nitrate sensing during dormancy cycling.
