# Supplementary figures and images for: Temperature, light and nitrate sensing coordinate Arabidopsis seed dormancy cycling, resulting in winter and summer annual phenotypes
Source: Plant J. 2013 Apr 17;74(6):1003–15. doi: 10.1111/tpj.12186 (PMC3764396; doi:10.1111/tpj.12186)

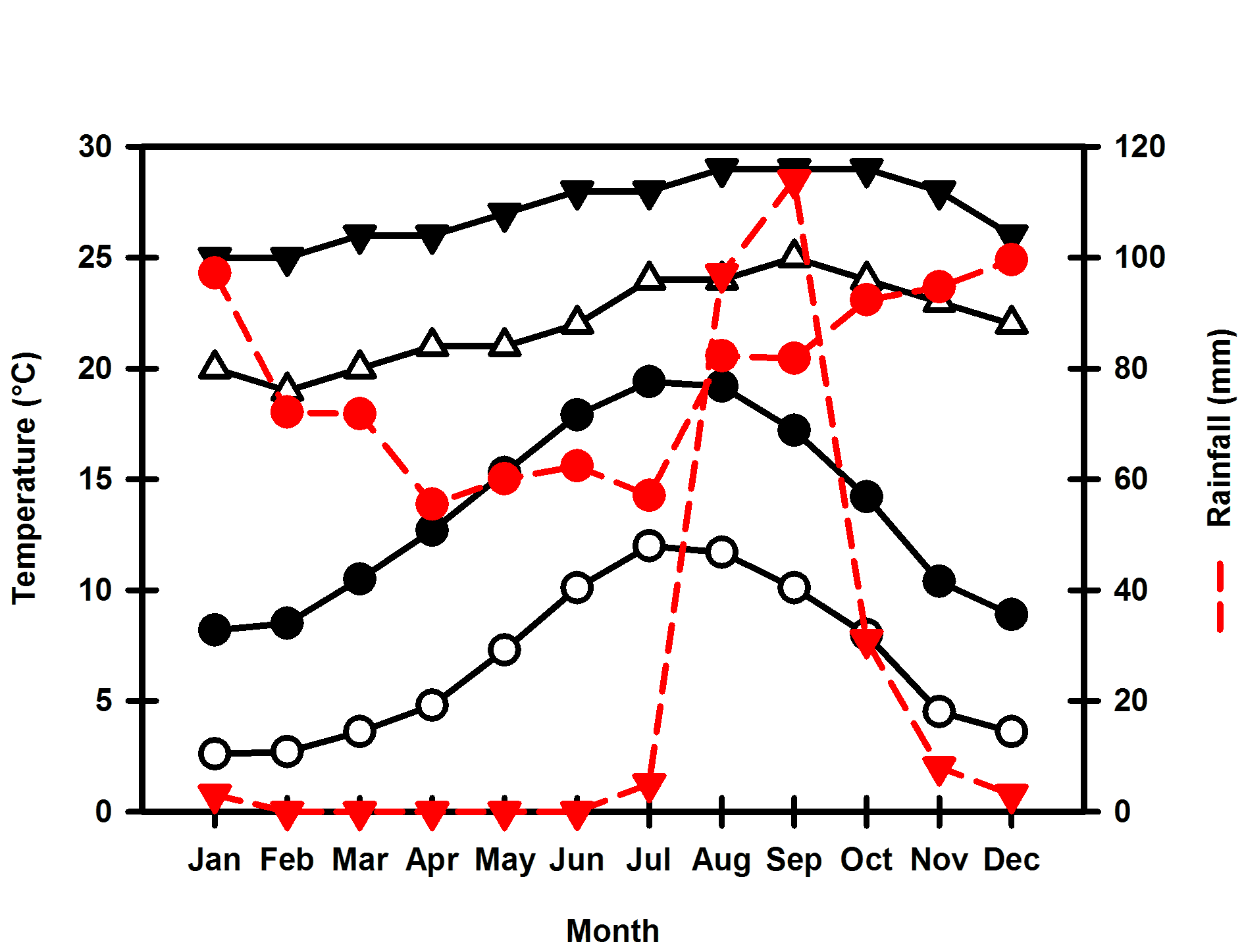

Supplement: Supplementary file 1 [file tpj0074-1003-SD1.tif]

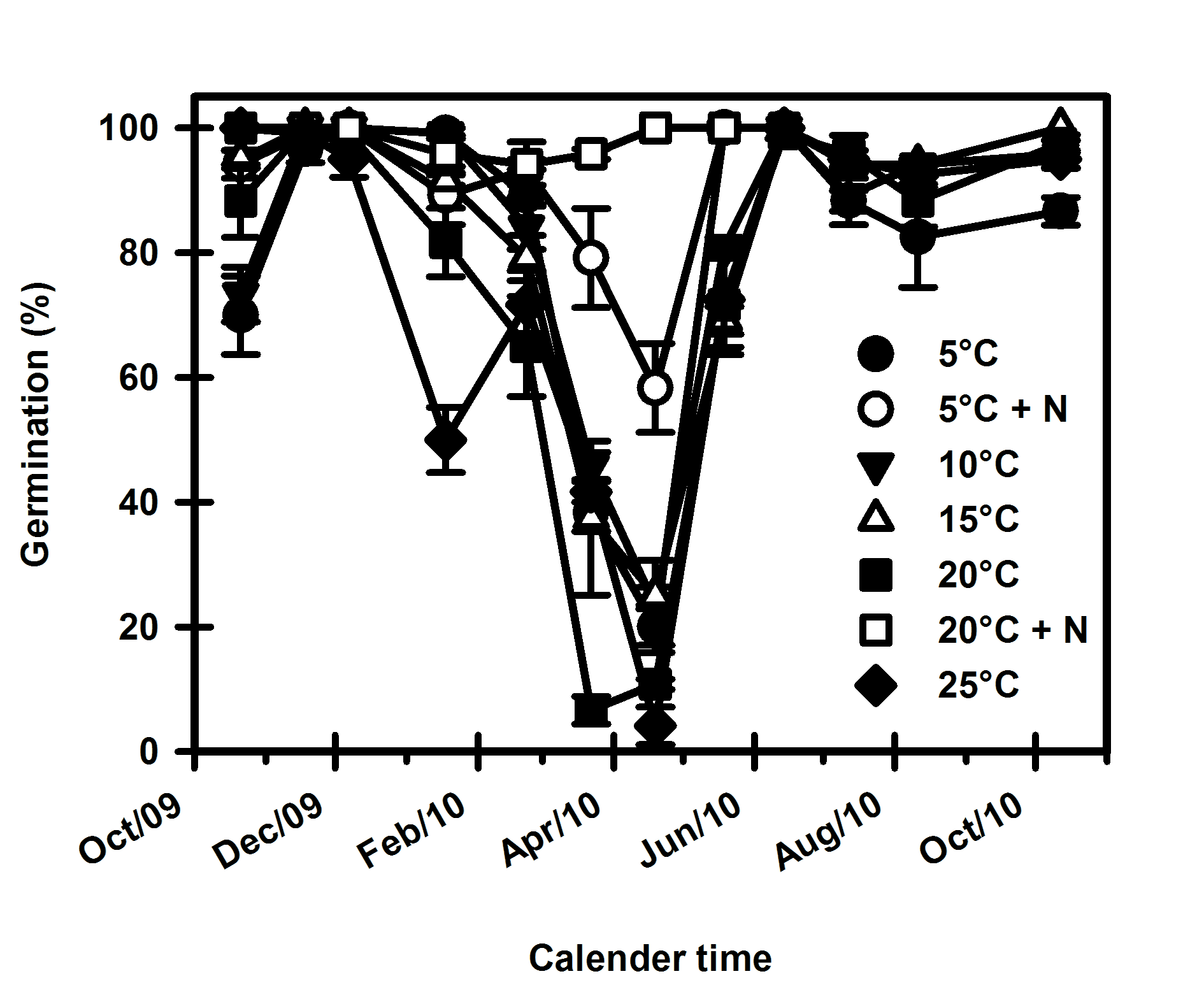

Supplement: Supplementary file 2 [file tpj0074-1003-SD2.tif]

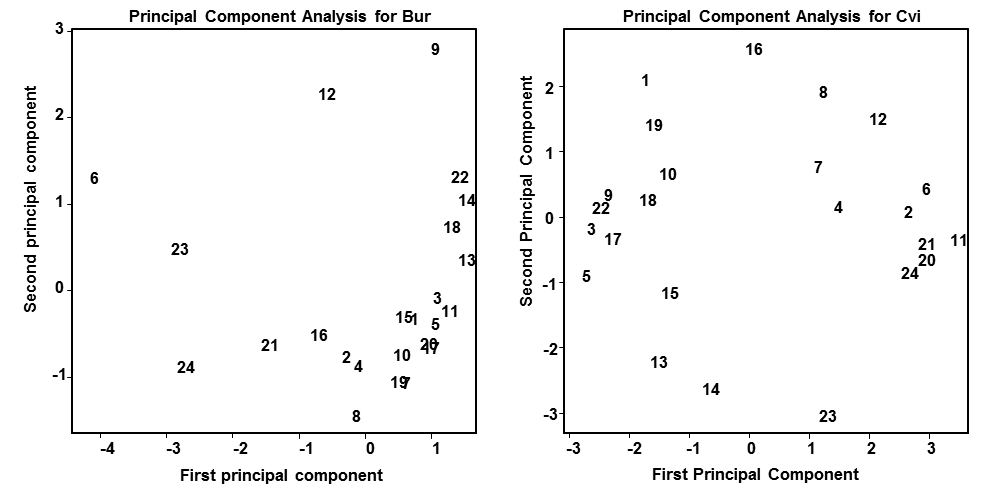

Supplement: Supplementary file 3 [file tpj0074-1003-SD3.tif]
