# Supplementary material for: Temperature, light and nitrate sensing coordinate Arabidopsis seed dormancy cycling, resulting in winter and summer annual phenotypes
Source: Plant J. 2013 Apr 17;74(6):1003–15. doi: 10.1111/tpj.12186 (PMC3764396; doi:10.1111/tpj.12186)
Supplement: Supplementary file 4 [file tpj0074-1003-SD4.docx]

| **Table S1: Probe sets for genes of interest used for Nanostring analysis** | | | |  | |  |
| --- | --- | --- | --- | --- | --- | --- |
| Gene symbol | Accession number | Targeted Region | Target Sequence |  | | |
| ABI2 | NM_125087.2 | 1154-1254 | GCTCATGCTCCGGAAACTGTTGGGTCTACCTCGGTGGTTGCGGTTGTCTTTCCGACTCACATCTTTGTCGCGAATTGCGGCGACTCTAGGGCGGTTTTGT | | | |
| *ABI3* | NM_113376.3 | 801-901 | GTCTTCAACATCTCCAGCTCCTGTCAACGCAATCGTCTCCTCAGCCTCTTCTTCTTCGGCAGCTTCTTCCTCCACTTCCTCAGCTGCTTCTTGGGCTATA | | | |
| *ABI4* | NM_129580.1 | 725-825 | GCAGGAACAAGGAGGAAGTGGGTGTAATAATAATAGTTCGATGGAGGATTTGAACTCTCTAGCTGGTTCGGTGGGTTCGAGTCTATCAATAACTCATCCA | | | |
| *ABI5* | NM_129185.3 | 513-613 | TGGTGTTGGTGTCTTTAGTGGTGGTTCTAGAGGCAACGAAGATGCTAACAATAAGAGAGGGATAGCGAACGAGTCTAGTCTTCCTCGACAAGGCTCTTTG | | | |
| *CYP707A2* | NM_128466.2 | 255-355 | AGAGACACTCCGCCTCTACACAGAAAATCCCAATTCCTTCTTCGCCACTCGCCAAAACAAGTACGGGGATATATTCAAGACGCACATATTAGGATGTCCA | | | |
| *DOG1* | NM_123951.2 | 599-699 | CGACAAGCAAGAAGAAGCTATGGCTCGTTTATTGGTCGAGGCCGATAATCTAAGGGTTGATACTTTAGCGAAGATCCTCGGGATTCTATCTCCGGTACAA | | | |
| *FLC* | NM_001161231.1 | 122-222 | ATCCGTCGCTCTTCTCGTCGTCTCCGCCTCCGGCAAGCTCTACAGCTTCTCCTCCGGCGATAACCTGGTCAAGATCCTTGATCGATATGGGAAACAGCAT | | | |
| *Ga2ox2* | NM_102743.2 | 464-564 | TCAATGCTAATCCTCAGCTCTCCTCTCCTAAAACCTCCGCCGTTTTCCGTCAAACCCCTCAAATTTTCCGTGAGTCGGTGGAGGAGTACATGAAGGAGAT | | | |
| *Ga3ox1* | NM_101424.2 | 320-420 | GGGGTGCCTTCCAAATCTCAAACCACGGCGTGCCTTTGGGACTTCTCCAAGACATTGAGTTTCTCACCGGTAGTCTCTTCGGGCTACCTGTCCAACGCAA | | | |
| *GID1A* | NM_111384.3 | 290-390 | TCAAAGTAGCCTACAATATCCTTCGTCGCCCTGATGGAACCTTTAACCGACACTTAGCTGAGTATCTAGACCGTAAAGTCACTGCAAACGCCAATCCGGT | | | |
| *MFT* | NM_101672.3 | 309-409 | CCGAACATGAGAGAATGGGTCCACTGGATTGTCGTGGATATTCCCGGAGGCACAAATCCCTCAAGAGGAAAAGAGATACTTCCATACATGGAACCAAGGC | | | |
| *NCED6* | NM_113327.2 | 1334-1434 | AACCGTAAGGTTATCGTAACCGGAGTGAATTTAGAAGCGGGTCACATAAACCGTAGTTACGTGGGCCGGAAAAGCCAGTTCGTTTACATAGCAATAGCCG | | | |
| *NR1* | NM_106425.2 | 513-613 | CTCCATTACGTCCGCAACCACGGTGCAGTTCCCAAAGCGAATTGGTCAGACTGGTCAATCGAAATTACCGGACTCGTTAAACGTCCGGCTAAATTCACCA | | | |
| *NRT1.1* | NM_101083.3 | 1505-1605 | CCCACAATATCTTATTGTCGGTATCGGCGAAGCGTTAATCTACACAGGACAGTTAGATTTCTTCTTGAGAGAGTGCCCTAAAGGTATGAAAGGGATGAGC | | | |
| *PIL5* | NM_179665.2 | 520-620 | ATTTCTCGAGGCTGAGAGGGGATTTTAATAACGGTAGAGGTGGTGAATCTGGACCGTTGCTTTCGAAGGCGGTTGTGAGAGAATCTACGCAGGTAAGTCC | | | |
| *PYL7* | NM_116332.3 | 328-428 | TATAAGCAGATGCACTGTAAATGGTGATCCTGAGATCGGTTGTCTCAGAGAAGTAAATGTCAAATCTGGTCTTCCAGCAACCACCAGTACAGAGAGATTG | | | |
| *PYR1* | NM_117896.2 | 601-701 | TTGTGAAGCTTAATTTGCAGAAACTCGCGACGGTTGCTGAAGCTATGGCTCGTAACTCCGGTGACGGAAGTGGTTCTCAGGTGACGTGAAAATGAAGAAA | | | |
| *RGA2* | NM_101361.2 | 39-139 | AGAAGTGGTAGTGGAGTGAAAAAACAAATCCTAAGCAGTCCTAACCGATCCCCGAAGCTAAAGATTCTTCACCTTCCCAAATAAAGCAAAACCTAGATCC | | | |
| *RGL2* | NM_111216.2 | 1068-1168 | GTCGTTTCGTCTCACCGGAATCGGACCACCGCAGACGGAGAATTCAGATTCGCTTCAACAGTTAGGTTGGAAATTAGCTCAATTCGCTCAGAACATGGGC | | | |
| *Snrk2.1* | NM_120946.4 | 1315-1415 | CACTCCTTGGTGAGTTGGTGACTTGCTTCTGGTAAAAAGAAAAACACATTTGTCAAAGCCAAAGGCTGTAAACTACGTGCTGGTTGTTTCACTTTTAGAT | | | |
| *Snrk2.4* | NM_100969.3 | 1242-1342 | AAAGAGAACCCAACCTTCTCCCTTCAGACCGTTGAAGAGATCATGAAGATAGTGGCTGACGCCAAAACACCGCCTCCTGTTTCCCGATCCATCGGAGGTT | | | |
| *SPT* | NM_119857.2 | 1083-1183 | TGGTCATTCCAACGCAAACATAACCGGGGAACAAGCTCTGTTTGATGGACAACCTGACCTAAAAGATCGAATTACTTGAACAGTGTCCCAACTTCGGGAT | | | |
| *TIP41like* | NM_119592.4 | 768-868 | TATAGGTTTGGCGAAGATGAGGCACCAACTGTTCTTCGTGAAAACTGTTGGAGAGAAGCAACATTTCAGTCTCTATCTGCGAAAGGGTATCCAGTTGACT | | | |
|  |  |  |  |  | |  |
| **TRANSCRIPT VARIANT DATA** | |  |  |  | |  |
| Target Accession | | Gene symbol | TVs Hit | Other TVs giving signal |  |  |
| NM_125087.2:1154 | | *ABI2* | 2 | NM_001125976.1 |  |  |
| NM_102743.2:464 | | *GA2OX2* | 2 | NM_001036035.1 |  |  |
| NM_001161231.1:122 | | *FLC* | 3 | NM_121052.2;NM_001085094.1 |  |  |
| NM_179665.2:520 | | *PIL5* | 2 | NM_127577.3 |  |  |
