# Supplementary material for: Temperature, light and nitrate sensing coordinate Arabidopsis seed dormancy cycling, resulting in winter and summer annual phenotypes
Source: Plant J. 2013 Apr 17;74(6):1003–15. doi: 10.1111/tpj.12186 (PMC3764396; doi:10.1111/tpj.12186)
Supplement: Supplementary file 5 [file tpj0074-1003-SD5.docx]

**Experimental procedures**

**Seed production:** Bur and Cvi seeds were produced in a heated glass house from May to July 2009, with a mean day/night temperature alternation of 22.3°C/17.4°C. Nondormant seeds of the *Arabidopsis thaliana* ecotype Burren (Bur-0) or Cape Verdi Island (Cvi) were sown into compost (Levingtons F2/sand /vermiculite at a ratio of 6/1/1) in multicell trays held in capillary matting lined seed trays. When germinated seeds had established the number of seedlings was reduced to one per cell. Upon bolting each plant was covered with an Aracon (Betatech bvba, Belgium). Upon completion of seed maturation seeds were harvested by hand threshing and cleaned seeds equilibrated at 55% RH/ 20°C for 7 days to produce an equilibrium moisture content of 6-10 % on a dry weight basis. Seeds were then stored at -80°C.

**Seed burial:** Seed mortality was reduced by dressing with Metalaxyl (Hockley International, UK) at 1 g active fungicide/ kg seeds. Seeds were dispersed in Ballotini balls (0.1-0.25 mm diameter) (Potters Ballotini Ltd, UK) within the range of particle sizes of the sandy loam soil in the field trial area. Seeds were dispersed at a density of 40 seeds /g of Ballotini balls to further reduce seed mortality ([Van Mourik *et al.* 2005](#_ENREF_12)). For physiological analysis 140 mg of seeds were dispersed in 85 g of Ballotini balls placed in a nylon bag (10 x 10 cm) constructed from 125 μm nylon mesh (Clarcor UK, UK) and sealed with a WeLoc® bag clip (size PA110) (WeLoc - Weland M. AB, Sweden). One bag was prepared for each month of the time course. For molecular analysis of dormancy status four bags were prepared for each month of the time course each with 50 mg of seeds dispersed in 28 g of Ballotini balls contained in a nylon bag (7 x 7 cm) sealed with a PA70 bag clip. Each bag was identified with a plastic label attached to each clip with wire.

Seed bags were buried at a depth of 5 cm in a random plot design. Soil moisture sensors (SM200, Delta-T Devices Ltd, UK) and Thermistore temperature probes (Betatherm, Ireland) linked to a data logger (Delta-T Devices Ltd, UK) recorded soil moisture and temperature at seed depth within a dummy bag.

**Seed recovery:** Seed bags were recovered from the field monthly. Samples for molecular analysis were recovered from the field in the dark. A light proof box (H x W x L, 300 x 350 x 500 m had two circular arm holes 120 mm in diameter located 1/3 of the distance along the length, and 50 mm from the edge on the top. Long reach gauntlets were attached to these holes with retaining rings (Marigold Emperor ME106, Buck and Hickman, Coventry, UK). The box was placed over the burial site and the base sealed with soil to exclude light. The four molecular samples were then exhumed and placed in a laminated foil bag 11 x 24 cm (Moore and Buckle, St. Helens, UK) and sealed with a WeLoc® PA150 clip. On return to the laboratory seeds for molecular analyses were immediately separated from the Ballotini balls under a green safe light. Seed bags were removed from the light proof bag and each bag washed in cold water to remove soil. Ballotini balls and seeds from each bag were washed into separate 50-mL centrifuge tubes. Ballotini balls and seeds were allowed to settle, and the bulk of the water poured off. Ballotini balls and seeds were washed 3 times. Tubes were gently shake to migrate seeds over the Ballotini balls to the tube walls where they were removed with a pastette (3 mL)(Jencons Ltd, UK) to 2 mL Eppendorf tubes, frozen in liquid nitrogen and stored at -80°C. Seeds for physiological analysis were separated from Ballotini balls in a glass beaker in the light in a similar fashion as above and immediately used for dormancy testing. Further details of seed production, burial and recovery can be found in Footitt and Finch-Savage ([Footitt and Finch-Savage 2011](#_ENREF_8)).

**Dormancy testing:** Seeds recovered for physiological analysis were surface-sterilize in a 0.125 % sodium hypochlorite solution (Household bleach (5% sodium hypochlorite) diluted to 2.5%) for 5 min then washed three times in water.

Using an Ultipette™ BARKY CP-100 tips (Barkey Instruments International, UK) 50 seeds were plated out in triplicate plates (124 x 88 x 22 mm) (Stewart Plastics Ltd, UK). Each plate contained two sheets of Whatman 3MM chromatography paper and 8 mL of the appropriate solution. Plates were placed in sealable freezer bags and incubated at 20°C under continuous light and germination scored at 2-3 day intervals for up to 28 days.

Thermodormancy was tested by incubating seeds on water as above at 5, 10, 15, 20, and 25°C. Nitrate sensitivity was tested by incubating seeds in 10 MM KNO_3_ as above. Seed viability was at 20°Cin the light using 100 μM GA/ 50 μM Fluridone in citrate /phosphate buffer (pH 5.0) as above. Fresh Bur and Cvi seeds were also incubated in 75 x 75 mm plates (Stewart Plastics Ltd, UK) on a sheet of 3MM chromatography paper on 0.7% water agarose in continuous light on a thermogradient table (Grant Instruments, UK) over a temperature range of 5-31 ^o^C. Germination was scored as emergence of the radicle through the testa.

**Hydrothermal time analysis:** Depth of dormancy at each harvest was evaluated using a hydrothermal time approach ([Bradford 2002](#_ENREF_1), [Finch-Savage *et al.* 2000](#_ENREF_6)) shown for a given seed in the population in equation 1.

Equation 1: *HTT* = (*Ψ* - *Ψ_b_*) (*T - T_b_*) *t*

Here time to germination (*t*) is a function of the extent ambient temperature (*T*) and water potential (*Ψ*) exceeds a base temperature (*T_b_*) and *Ψ_b_* respectively, below which germination does not progress to completion. HTT and *T_b_* can be considered as constants so the only variable parameter *Ψ_b_* determines the germination rate of the seed. Therefore as *Ψ_b_* becomes less negative, HTT accumulates more slowly, germination rate decreases and dormancy increases. When *Ψ_b_* rises above ambient *Ψ* (saturated soil ≤ 0 MPa) germination no longer proceeds to completion.

Hydrothermal time parameters were determined from cumulative germination data collected at 5-30°C as above and at 10°C and 20°C at a range of water potentials (-0.1. -0.2, -0.3, -0.4, -0.5, -0.6 MPa) using 25 ml of PEG 8000 solution held in germination plates as above. The additional liquid reservoir was accommodated by placing seeds on 125 μm nylon mesh (Clarcor UK, UK), on top of one sheet of Whatman 3MM chromatography paper, 1 mm nylon mesh, and a piece of glass-drying mat (Nisbits Ltd, UK). Seeds at each harvest were tested at the above water potentials at 15°C in the light.

The *Ψ_b_* of “non-dormant” seeds was determined with seeds dry after-ripened for 145 days and assumed to have minimal dormancy. The resulting hydrothermal time parameters for these after-ripened seeds were HTT, 29.714, *T_b_,* 2.208°C, and mean *Ψ_b_*, -0.759 MPa. HTT analysis for seeds prior to burial and following recovery from the field used the HTT and *T_b_* derived for after-ripened seeds to calculate mean *Ψ_b_* for each harvest.

**Seedling emergence:** Seeds were buried in the field on 2/11/2009 and emergence tests carried out as described previously by thoroughly stirring the soil at seed depth in each pot monthly ([Footitt *et al.* 2011](#_ENREF_7)).

**Seed nitrate:** The nitrate content of seeds was determined by a steam distillation method using a FOSS FIAstar 5000 Flow Injection Analyser for end point determination ([Bremner and Keneney 1965](#_ENREF_2)).

**Gene expression analysis:** RNA was extracted using nuclease free reagents using the methods of Xu et al., and Gehrig et al., ([Gehrig *et al.* 2000](#_ENREF_9), [Xu *et al.* 2010](#_ENREF_13)) adapted for seeds. Seeds for time zero that had been imbibed for 24 hours in the dark at 20°C and seeds recovered from the field (≤125 mg) were homogenised from frozen in 0.9 ml preheated extraction buffer (65°C) (100 mM TRIS-HCl pH 8.0, 25 mM EDTA, 2 M NaCl, 2% W/V CTAB, 2 % PEG 20,000 and 2% v/v β-mercaptoethanol). Samples were then incubated at 65°C for 15 mins. 0.5 ml of chloroform was added to each sample, briefly vortexed and centrifuged at 12,000 rpm/ 4°C/10 min. The supernatant was transferred to a new tube and 133 µl of 5M NaCl and 0.4 ml of chloroform added, mixed and re- centrifuged. This last step was then repeated and RNA precipitated for the supernatant by adding ½ volume of isopropanol and ½ volume of 1.2M NaCl/0.8M tri-Na citrate dehydrate mixing gently and incubating at room temperature for 15 minutes. RNA was recovered by centrifugation at 12,000 rpm/ 4°C/10 min and the pellet washed in 75% ethanol, re- centrifuged and the pellet air dried for 10 minutes and dissolved in 120 µl of water and re-precipitated with a ½ volume of 5M NH4oAc and 2.5 volumes of ethanol at -20°C for at least one hour. RNA was recovered following the first precipitation and dissolved in 40 µl water.

Gene expression was analyzed using the Nanostring ncounter gene expression system ([Geiss *et al.* 2008](#_ENREF_10)) by UCL Genomics, University College London using 100 ng of total RNA. The genes included those involved in abscisic acid (ABA) and gibberellic acid (GA) biosynthesis (*NINE-CIS-EPOXYCAROTENOID DIOXYGENASE 6 (NCED6)*, *GIBBERELLIN 3 BETA-HYDROXYLASE 1* (*GA3ox1*) and catabolism (*CYTOCHROME P450, 707A2 (CYP707A2), GIBBERELLIN 2-OXIDASE 2 (GA2ox2*)), ABA signaling *(PYRABACTIN RESISTANCE 1(PYR1), PYR1-LIKE 7 (PYL7), ABA INSENSITIVE 2 (ABI2),* *SNF1-RELATED PROTEIN KINASE 2.1 (SnrK2.1)* and *SNF1-RELATED PROTEIN KINASE 2.4 (SnrK2.4), ABA INSENSITIVE 3 (ABI3), ABA INSENSITIVE 4 (ABI4),* and *ABA INSENSITIVE 5 (ABI5))*, GA signalling *(GA INSENSITIVE DWARF1A (GID1A), RGA-LIKE 2 (RGL2),* *GIBBERELLIC ACID INSENSITIVE RESTORATION ON GROWTH ON AMONIA 2 (RGA2)) ,* and members of the Phytochrome Interacting Factor (PIF) family *(PHYTOCHROME INTERACTING FACTOR 3-LIKE 5 (PIL5)* and *SPATULA (SPT)).* Other genes investigated include: *DOG1*, *FLOWERING LOCUS C (FLC), MOTHER OF FT (MFT), NRT1.1* and *NITRATE REDUCTASE 1 (NR1).* The Nanostring probes targeting the genes *ABI2 (*At5g57050), *ABI3 (*At3g24650), *ABI4* (At2g40220), *ABI5* (At2g36270), *CYP707A2* (At2g29090), *DOG1* (At5g45830), *FLC* (At5g10140), *Ga2ox2* (At1g30040), *Ga3ox1* (At1g15550), *GID 1A* (At3g05120), *MFT* (At1g18100), *NCED6* (At3g24220), *NR1* (At1g77760), *NRT 1.1* (At1g12110), *PIL5* (At2g20180), *PYL7* (At4g01026), *PYR1* (At4g17870), *RGA2* (At1g14920), *RGL2* (At3g03450), *Snrk 2.1* (At5g08590), *Snrk 2.4* (At1g10940), *SPT* (At4g36930), and the internal control gene *TIP41-like* (At4g34270) ([Footitt, et al. 2011](#_ENREF_7)) were designed and synthesized by NanoString Technologies (http://www.nanostring.com) (Table S1). The mRNA counts for the genes under investigation were normalized to the Nanostring internal reference and the reference gene *TIP41-like* as described in the nCounter Gene Expression Assay Manual (http://www.nanostring.com/uploads/Manual_Gene_Expression_Assay.pdf/). The reference gene At4g34270 (*Tip41-like*) was selected for normalization of the data due to its stability of expression in our seed dormancy microarray data ([Cadman *et al.* 2006](#_ENREF_3), [Finch-Savage *et al.* 2007](#_ENREF_5)) where it has the lowest fold change in expression (maximum 1.5; Dataset S3) and coefficient of variation across 11 different hydrated dormancy states when compared with others evaluated as reference genes in plants ([Remans *et al.* 2008](#_ENREF_11)). It was also identified as one of the most stable reference genes for use in expression analysis by QPCR in Arabidopsis seeds ([Dekkers *et al.* 2012](#_ENREF_4)). In the Nanostring data, *TIP41-like* shows a maximum fold change of 1.7 across all harvests in the field with no discernible pattern of expression. QPCR also shows it to be relatively stable with no discernable pattern during dormancy cycling in the field (maximum fold change in Cvi 2.5: in Bur 2.4).

Gene expression of *CIPK23* (At1g30270) and *PHYA (*At1g09570*)* using the primers (*CIPK23* Forward 3’-GAAAACCATCGACGAAGAGAA-5’, Reverse 3’- GGTTAGCCATGGCACCATTA-5’; *PHYA* Forward 3’-AAGATCTGATCATGGCTTCTTGA-5’, Reverse 3’- GGCCTAGAGCCTGACATTTTT-5’) was determined by QPCR of cDNA at a dilution of 1/25 as described previously ([Footitt, et al. 2011](#_ENREF_7)).

**Statistical analyses of Gene Expression profile data, measures of dormancy and field temperatures in Bur and Cvi**

**Hierarchical cluster analysis:** Pearson correlation coefficients between all pairs of genes were calculated for the gene expression data for the 24 genes considered at 11 time points (Bur) and 12 time points (Cvi) for each ecotype separately. The correlation coefficients were transformed to measures of similarity by adding one to each value and dividing the resulting value by two, so that genes with a correlation of +1 would have a similarity of 1, those with a correlation of -1 would have a similarity of 0, and those with a correlation of 0 would have a similarity of 0.5. The resulting matrices of similarity measures were then subjected to a hierarchical cluster analysis using the complete link (furthest neighbour) algorithm, and the results displayed using a dendrogram (Figure 7). An assessment of the level of agreement between the matrices of similarities for Bur and Cvi gene expression data was obtained using a Mantel test based on product-moment correlations with 1000 permutations.

**Principal component analysis** was applied to the gene expression data for each ecotype separately, considering the set of observations across the 24 genes at each observation time as the original variates, and the 24 genes as the observational units, in each case. Plots of the scores (for the 24 genes) on the first three principal components were produced to identify groupings of genes with similar expression profiles across the observation times. A Procrustes Rotation analysis was used to assess the similarity of the configurations of the 24 genes on the first three principal components from the analysis of the data for each ecotype (Figure S3). This analysis rotates and reflects the configuration for one ecotype to find the closest fit of all points to the equivalent points in the configuration for the other ecotype. Where the points for a particular gene in the two configurations are close together in the consensus plot does not necessarily indicate that the genes have the same profiles over time, but that the associations between the sets of genes that have similar locations for the two configurations are similar, while those genes with very different locations for the two configurations have different associations with other genes for the two ecotypes.

Further principal component analyses were applied to the gene expression data for each ecotype separately, to identify the genes primarily contributing to the variation in the profiles of gene expression values at each observation time (with the set of observations for each gene now being considered as the original variates, and the 11 or 12 observation times as the observational units). Biplots were produced to show the scores for each observation time on each of the principal axes, together with vectors representing the contributions of each gene to these principal components. This form of presentation allows identification of genes with higher and lower expression values at particular observation times.

Linear regression analyses were used to assess the relationship between the principal components from these latter analyses and measures of the field temperature prior to the recovery of seeds from the field, and to assess the relationship between measures of dormancy (*Ψ_b_* for ecotype Burren, AR50 for ecotype Cvi) and the principal components.

All analyses were performed using the GenStat program (Thirteenth Edition).

**Bradford, K.J.** (2002) Applications of hydrothermal time to quantifying and modeling seed germination and dormancy. *Weed Science*, **50**, 248-260.

**Bremner, J.M. and Keneney, D.R.** (1965) Steam distillation methods for determination of ammonium, nitrate and nitrite. *Analytica Chimica Acta*, **32**, 9.

**Cadman, C.S.C., Toorop, P.E., Hilhorst, H.W.M. and Finch-Savage, W.E.** (2006) Gene expression profiles of Arabidopsis Cvi seeds during dormancy cycling indicate a common underlying dormancy control mechanism. *Plant Journal*, **46**, 805-822.

**Dekkers, B.J.W., Willems, L., Bassel, G.W., van Bolderen-Veldkamp, R.P., Ligterink, W., Hilhorst, H.W.M. and Bentsink, L.** (2012) Identification of Reference Genes for RT-qPCR Expression Analysis in Arabidopsis and Tomato Seeds. *Plant and Cell Physiology*, **53**, 28-37.

**Finch-Savage, W.E., Cadman, C.S.C., Toorop, P.E., Lynn, J.R. and Hilhorst, H.W.M.** (2007) Seed dormancy release in Arabidopsis Cvi by dry after-ripening, low temperature, nitrate and light shows common quantitative patterns of gene expression directed by environmentally specific sensing. *Plant Journal*, **51**, 60-78.

**Finch-Savage, W.E., Phelps, K., Peach, L. and Steckel, J.R.A.** (2000) Use of threshold germination models under variable field conditions. In *Seed Biology: Advances and Applications* (Black, M., Bradford, K.J. and VazquezRamos, J. eds), pp. 489-497.

**Footitt, S., Douterelo-Soler, I., Clay, H. and Finch-Savage, W.E.** (2011) Dormancy cycling in Arabidopsis seeds is controlled by seasonally distinct hormone-signaling pathways. *Proceedings of the National Academy of Sciences of the United States of America*, **108**, 20236-20241.

**Footitt, S. and Finch-Savage, W.E.** (2011) 'Production of Seed Samples for the Effective Molecular Analysis of Dormancy Cycling in Arabidopsis In *Seed Dormancy. Methods in Molecular Biology* (Kermode, A. ed: Springer, pp. 65 - 79.

**Gehrig, H.H., Winter, K., Cushman, J., Borland, A. and Taybi, T.** (2000) An improved RNA isolation method for succulent plant species rich in polyphenols and polysaccharides. *Plant Molecular Biology Reporter*, **18**, 369-376.

**Geiss, G.K., Bumgarner, R.E., Birditt, B., Dahl, T., Dowidar, N., Dunaway, D.L., Fell, H.P., Ferree, S., George, R.D., Grogan, T., James, J.J., Maysuria, M., Mitton, J.D., Oliveri, P., Osborn, J.L., Peng, T., Ratcliffe, A.L., Webster, P.J., Davidson, E.H. and Hood, L.** (2008) Direct multiplexed measurement of gene expression with color-coded probe pairs. *Nature Biotechnology*, **26**, 317-325.

**Remans, T., Smeets, K., Opdenakker, K., Mathijsen, D., Vangronsveld, J. and Cuypers, A.** (2008) Normalisation of real-time RT-PCR gene expression measurements in Arabidopsis thaliana exposed to increased metal concentrations. *Planta*, **227**, 1343-1349.

**Van Mourik, T.A., Stomph, T.J. and Murdoch, A.J.** (2005) Why high seed densities within buried mesh bags may overestimate depletion rates of soil seed banks. *Journal of Applied Ecology*, **42**, 299-305.

**Xu, J., Aileni, M., Abbagani, S. and Zhang, P.** (2010) A Reliable and Efficient Method for Total RNA Isolation from Various Members of Spurge Family (Euphorbiaceae). *Phytochemical Analysis*, **21**, 395-398.
