# Supplementary material for: Temperature, light and nitrate sensing coordinate Arabidopsis seed dormancy cycling, resulting in winter and summer annual phenotypes
Source: Plant J. 2013 Apr 17;74(6):1003–15. doi: 10.1111/tpj.12186 (PMC3764396; doi:10.1111/tpj.12186)
Supplement: Supplementary file 8 [file tpj0074-1003-SD8.docx]

**Appendix S4: A review of the influence of nitrate on dormancy cycling**

It is well known that in the laboratory nitrate influences dormancy and the completion of germination in Arabidopsis and a wide range of annual species ([Footitt and Cohn 2001](#_ENREF_10), [Finch-Savage and Leubner-Metzger 2006](#_ENREF_9)). In the present work we consider the evidence that nitrate has a crucial influence on the annual dormancy cycle in the soil and the mechanism involved.

***Soil nitrate concentration:*** Nitrate concentration in soil solution fluctuates and can vary from almost 0 to 50 mmol l^-1^ (([Bouwmeester *et al.* 1994](#_ENREF_4)) and references therein), covering the range shown to elicit a response from seeds in the laboratory. Detailed investigations on the influence of nitrate on secondary dormancy and the annual dormancy cycle has been almost exclusively carried out in *Sisymbrium officinale* and Arabidopsis and so for brevity we will only report this work. In several studies annual variations in soil nitrate ([Bouwmeester and Karssen 1993](#_ENREF_5), [Derkx and Karssen 1993](#_ENREF_7)) and seed nitrate content ([Derkx and Karssen 1993](#_ENREF_7)) have been recorded. However, field experiments have also shown that dormancy patterns remain the same in seeds that are exposed to variation in soil nitrate and where it is not ([Bouwmeester and Karssen 1993](#_ENREF_5), [Derkx and Karssen 1993](#_ENREF_7), [Bouwmeester, et al. 1994](#_ENREF_4)). They conclude that changes in dormancy are driven by temperature and not influenced by soil moisture and soil nitrate. The same conclusion was also reached in studies with Arabidopsis where seeds were exposed to annual cycles of temperature on absorbent paper ın the dark showing seasonal dormancy patterns were mainly regulated by changes in sensitivity to light with some contribution from sensitivity to GA ([Derkx and Karssen 1994](#_ENREF_8)). However, it has been suggested that Arabidopsis seeds in these experiments were relatively unresponsive to nitrate because of the conditions under which they were produced, which affected their endogenous nitrate concentration ([Alboresi *et al.* 2005](#_ENREF_1)). They showed that the accumulation of nitrate in seeds leads to lower dormancy in Arabidopsis; this does not affect the onset of dormancy, but rather its maintenance ([Alboresi, et al. 2005](#_ENREF_1)). This effect is the same in *S. officinale* where there was a linear relationship between seed nitrate content and germination in the dark ([Bouwmeester, et al. 1994](#_ENREF_4)). However, in the latter work it was observed that these differences in nitrate content were lost through leaching when seeds were buried in the soil. Hilhorst ([Hilhorst 1990b](#_ENREF_15)) showed most endogenous nitrate is leached from seeds in the first 24 h of imbibition on water in the laboratory. Thus high nitrate content will relieve dormancy, but only temporally when placed in soil; therefore it was suggested endogenous nitrate concentration has little ecological importance ([Bouwmeester, et al. 1994](#_ENREF_4)). In contrast, seed sensitivity to nitrate is likely to have a significant ecological role in response to soil nitrate that varies over the year, but can also inform about the presence of plants and gap sensing at the appropriate time of year.

***Sensitivity to nitrate:*** In *S. officinale*, inhibitors of nitrate reductase activity did not influence nitrate-stimulated germination in the laboratory, suggesting nitrate has a direct regulatory role ([Hilhorst and Karssen 1989](#_ENREF_17)). Additionally, nitrate and light acted synergistically ([Hilhorst and Karssen 1988](#_ENREF_16)), wıth endogenous nitrate a limiting factor in light induced germination. In the field, Derkx and Karssen ([Derkx and Karssen 1993](#_ENREF_7)) showed that endogenous levels of nitrate in seeds may vary due to seasonal influences, but they do not link these changes to shifts in the nitrate dose response curves. They suggest a model where temperature results in reversible changes in sensitivity to light and nitrate, which occur at the level of receptors and control via the availability of receptors is likely. This is consistent with the earlier conclusions of Hilhorst ([Hilhorst 1990b](#_ENREF_15)) in the laboratory studying secondary dormancy. Similarity in fluence-response curves and nitrate dose response led to the hypothesis that phytochrome and nitrate share the same site of action and that Pfr and nitrate bind to the same receptor. Thus formulations from the receptor occupancy theory can be used to describe the response to light and nitrate during the induction of secondary dormancy in *S. officinale* ([Hilhorst 1990a](#_ENREF_14), [b](#_ENREF_15)). In support of this in Arabidopsis, nitrate also promotes germination by reducing the light requirement ([Hilhorst and Karssen 1988](#_ENREF_16), [Batak *et al.* 2002](#_ENREF_3)).

***Role of NRT1.1:*** Alboresi et al. ([Alboresi, et al. 2005](#_ENREF_1)) suggest that the nitrate receptor hypothesized by Hilhorst ([Hilhorst 1990b](#_ENREF_15)) could be NRT1.1. Although molecular mechanisms involved in N sensing and signaling are still poorly understood, NRT1.1 appears to have a role as a dual-function transporter/sensor ([Vidal *et al.* 2010](#_ENREF_23)). Gojon et al. ([Gojon *et al.* 2011](#_ENREF_12)) argue that NRT1.1 is therefore a nutrient transceptor (duel nutrient transport/signaling function), as described for membrane proteins in yeast and animals. As described above nitrate availability in the soil dramatically fluctuates in time and it is suggested NRT1.1 is an important component of a sensing system for changes in local external nitrate concentrations ([Gojon, et al. 2011](#_ENREF_12)). Nitrate acts as a signal molecule that influences gene expression involved in many aspects of plant metabolism and development ([Krouk *et al.* 2010](#_ENREF_19)). NRT1.1 phosphorylation alters it from a low-affinity carrier to a high-affinity carrier ([Liu and Tsay 2003](#_ENREF_20)) and generates different levels of expression of primary nitrate response genes according to nitrate availability ([Vidal, et al. 2010](#_ENREF_23)). The ability of plants to sense nitrate, is a key process allowing these sessile organisms to adapt to nitrate fluctuations in the soil (([Gojon, et al. 2011](#_ENREF_12)) and refs therein) and this extends to seeds ([Alboresi, et al. 2005](#_ENREF_1)) as part of their essential role as environmental sensors to determine when to initiate the life cycle. This is a key step in determining the competitive advantage of an individual plant and species.

*NRT1.1* has a distinct expression pattern in our earlier laboratory dormancy cycling array data ([Cadman *et al.* 2006](#_ENREF_6)) (Data S3). *NRT1.1* had significantly higher expression in the imbibed dormant states (PD24, PD48, PD30 and SD1) and when seeds were exposed to light enabling the completion of germination (PDLN, LIG); the highest expression was in dormant seeds exposed to light that would not complete germination (PDL). Interestingly while *NRT1.1* expression increased progressively as primary dormant seeds remain in the imbibed state, expression of the only other nitrate transporter with a significant expression pattern *NRT2* expression declined.

In field experiments, both Cvi and Bur *NRT1.1* mRNA levels are very high in the dry seed and both ecotypes will respond to exogenous nitrate by breaking dormancy and completing germination if they are exposed to light. On burial, *NRT1.1* expression falls to low levels in both ecotypes, but unlike Cvi, Bur seeds remain sensitive to nitrate throughout the annual dormancy cycle. *NRT1.1* expression also follows a very distinct pattern rapidly increasing to five fold higher at maximum dormancy and continuing to rise to eight fold higher as dormancy is relieved to the point where only light is required to complete dormancy loss and progress to germination completion. In Bur the increase in *NRT1.1* expression is concurrent with increasing *MFT* expression and decreasing *DOG1* expression. Crucially, increased *NRT1.1* expression was followed by increased *CYP707A2* expression*.* The nitrate release of seed dormancy reported in Arabidopsis ([Alboresi, et al. 2005](#_ENREF_1)) acts by accelerating the decrease in ABA during germination ([Ali-Rachedi *et al.* 2004](#_ENREF_2)) via induction of the catabolic ABA gene CYP707A2 ([Matakiadis *et al.* 2009](#_ENREF_22)) as shown also for NO ([Liu *et al.* 2009](#_ENREF_21)). Alboresi et al, ([Alboresi, et al. 2005](#_ENREF_1)) question whether nitrate acts *per se* on seed germination or through the production of N-related signals.

As reported above, NRT1.1 is a dual affinity nitrate transporter, which can have a high or low affinity function depending on the phosphorylation status of threonine-101 (T101) ([Ho *et al.* 2009](#_ENREF_18)). In a low-nitrate condition T101 of NRT1.1 is phosphorylated by CBL-INTERACTING PROTEIN KINASE 23 (CIPK23) transforming it to a high affinity transporter, in high-nitrate condition the reverse happens. In seeds, we can speculate that when exogenous nitrate (high N) is applied to seed NRT1.1 will remain unphosphorylated with no requirement for CIPK23 i.e. the expression of *CIPK23* would be low. The response to this signal (high nitrate/low *CIPK23* expression) is to release dormancy and allow germination to reach completion. Intriguingly a comparison of changing depth of dormancy and the expression pattern of *CIPK23* shows clear correspondence in both Bur and Cvi. Initially following sowing *CIPK23* increases in both ecotypes (Figure 8c and 8e) as does depth of dormancy (Figure 8a and Footitt et al, ([Footitt *et al.* 2011](#_ENREF_11))). CIPK23 expression then declines first in Bur and then in Cvi in advance of dormancy release to a minimum when germination can occur (Figure 8b and 8d) and when seedling emergence was recorded in the field first for Bur and then Cvi (Figures 1c, 8d and 8b and Footitt et al ([Footitt, et al. 2011](#_ENREF_11)) respectively). In Cvi the seed also becomes sensitive to nitrate when CIPK23 expression is least (Figure 1b and Footitt et al. 2011). Thus the seed may be mimicking a high nitrate situation (relieves dormancy) to release dormancy via the same phosphorylation/dephosphorylation switch to elicit a downstream dormancy related response. This is possible since the nitrate transport activity is not required for the sensing function ([Ho, et al. 2009](#_ENREF_18)). In addition, this ion sensing mechanism for N-regulatory networks is also thought to have connections to other plant processes such as the circadian clock ([Gutierrez 2012](#_ENREF_13)). In the Bur ecotype *NRT1.1* expression also increases potentially increasing sensitivity to available nitrate compared to Cvi in line with its adaption to a N limiting environment. Thus local adaptation may have elaborated this particular dormancy related mechanism.

**Alboresi, A., Gestin, C., Leydecker, M.T., Bedu, M., Meyer, C. and Truong, H.N.** (2005) Nitrate, a signal relieving seed dormancy in Arabidopsis. *Plant Cell and Environment*, **28**, 500-512.

**Ali-Rachedi, S., Bouinot, D., Wagner, M.H., Bonnet, M., Sotta, B., Grappin, P. and Jullien, M.** (2004) Changes in endogenous abscisic acid levels during dormancy release and maintenance of mature seeds: studies with the Cape Verde Islands ecotype, the dormant model of Arabidopsis thaliana. *Planta*, **219**, 479-488.

**Batak, I., Devic, M., Giba, Z., Grubisic, D., Poff, K.L. and Konjevic, R.** (2002) The effects of potassium nitrate and NO-donors on phytochrome A- and phytochrome B-specific induced germination of Arabidopsis thaliana seeds. *Seed Science Research*, **12**, 253-259.

**Bouwmeester, H.J., Derks, L., Keizer, J.J. and Karssen, C.M.** (1994) Effects of endogenous nitrate content of Sisymbrium-officinale seeds on germination and dormancy. *Acta Botanica Neerlandica*, **43**, 39-50.

**Bouwmeester, H.J. and Karssen, C.M.** (1993) Annual changes in dormancy and germination in seeds of Sisymbrium-officinale (l) scop. *New Phytologist*, **124**, 179-191.

**Cadman, C.S.C., Toorop, P.E., Hilhorst, H.W.M. and Finch-Savage, W.E.** (2006) Gene expression profiles of Arabidopsis Cvi seeds during dormancy cycling indicate a common underlying dormancy control mechanism. *Plant Journal*, **46**, 805-822.

**Derkx, M.P.M. and Karssen, C.M.** (1993) Changing Sensitivity to Light and Nitrate but Not to Gibberellins Regulates Seasonal Dormancy Patterns in Sisymbrium-Officinale Seeds. *Plant Cell and Environment*, **16**, 469-479.

**Derkx, M.P.M. and Karssen, C.M.** (1994) Are Seasonal Dormancy Patterns in Arabidopsis-Thaliana Regulated by Changes in Seed Sensitivity to Light, Nitrate and Gibberellin. *Annals of Botany*, **73**, 129-136.

**Finch-Savage, W.E. and Leubner-Metzger, G.** (2006) Seed dormancy and the control of germination. *New Phytologist*, **171**, 501-523.

**Footitt, S. and Cohn, M.A.** (2001) Developmental arrest: from sea urchins to seeds. *Seed Science Research*, **11**, 3-16.

**Footitt, S., Douterelo-Soler, I., Clay, H. and Finch-Savage, W.E.** (2011) Dormancy cycling in Arabidopsis seeds is controlled by seasonally distinct hormone-signaling pathways. *Proceedings of the National Academy of Sciences of the United States of America*, **108**, 20236-20241.

**Gojon, A., Krouk, G., Perrine-Walker, F. and Laugier, E.** (2011) Nitrate transceptor(s) in plants. *Journal of Experimental Botany*, **62**, 2299-2308.

**Gutierrez, R.A.** (2012) Systems Biology for Enhanced Plant Nitrogen Nutrition. *Science*, **336**, 1673-1675.

**Hilhorst, H.W.M.** (1990a) Dose-response analysis of factors involved in germination and secondary dormancy of seeds of *Sisymbrium-officinale* .1. Phytochrome. *Plant Physiology*, **94**, 1090-1095.

**Hilhorst, H.W.M.** (1990b) Dose-response analysis of factors involved in germination and secondary dormancy of seeds of Sisymbrium-officinale .2. Nitrate. *Plant Physiology*, **94**, 1096-1102.

**Hilhorst, H.W.M. and Karssen, C.M.** (1988) Dual effect of light on the gibberellin-stimulated and nitrate-stimulated seed-germination of *Sisymbrium-officinale* and *Arabidopsis-thaliana*. *Plant Physiology*, **86**, 591-597.

**Hilhorst, H.W.M. and Karssen, C.M.** (1989) Nitrate reductase independent stimulation of seed-germination in *Sisymbrium-officinale l* (hedge mustard) by light and nitrate. *Annals of Botany*, **63**, 131-137.

**Ho, C.-H., Lin, S.-H., Hu, H.-C. and Tsay, Y.-F.** (2009) CHL1 Functions as a Nitrate Sensor in Plants. *Cell*, **138**, 1184-1194.

**Krouk, G., Crawford, N.M., Coruzzi, G.M. and Tsay, Y.F.** (2010) Nitrate signaling: adaptation to fluctuating environments. *Curr. Opin. Plant Biol.*, **13**, 266-273.

**Liu, K.H. and Tsay, Y.F.** (2003) Switching between the two action modes of the dual-affinity nitrate transporter CHL1 by phosphorylation. *Embo Journal*, **22**, 1005-1013.

**Liu, Y., Shi, L., Ye, N., Liu, R., Jia, W. and Zhang, J.** (2009) Nitric oxide-induced rapid decrease of abscisic acid concentration is required in breaking seed dormancy in Arabidopsis. *New Phytologist*, **183**, 1030-1042.

**Matakiadis, T., Alboresi, A., Jikumaru, Y., Tatematsu, K., Pichon, O., Renou, J.P., Kamiya, Y., Nambara, E. and Truong, H.N.** (2009) The Arabidopsis Abscisic Acid Catabolic Gene CYP707A2 Plays a Key Role in Nitrate Control of Seed Dormancy. *Plant Physiology*, **149**, 949-960.

**Vidal, E.A., Tamayo, K.P. and Gutierrez, R.A.** (2010) Gene networks for nitrogen sensing, signaling, and response in Arabidopsis thaliana. *Wiley Interdisciplinary Reviews-Systems Biology and Medicine*, **2**, 683-693.
