# Supplementary material for: Temperature, light and nitrate sensing coordinate Arabidopsis seed dormancy cycling, resulting in winter and summer annual phenotypes
Source: Plant J. 2013 Apr 17;74(6):1003–15. doi: 10.1111/tpj.12186 (PMC3764396; doi:10.1111/tpj.12186)
Supplement: Supplementary file 10 [file tpj0074-1003-SD10.docx]

**Figure S1:** **Annual weather patterns for the Burren, Ireland and Cape Verde Island.** Mean maximum and minimum monthly temperature and monthly rainfall. Burren data is the 30 year average data sets from Shannon Airport, the nearest Meteorological station (<http://www.burrenbeo.com/burren/climate>). Cape Verde Island data is for Praia, Cape Verdi (<http://www.bbc.co.uk/weather/3374333>). Burren °C Max., ●; Burren °C Min., **🌕**; Burren rain fall, ●; Cape Verdi °C Max., ▼; Cape Verdi °C Min., **△**; Cape Verdi rain fall, ▼.

**Figure S2: Changing thermodormancy in seeds recovered from the field**. Following recovery, seeds where incubated in the light at 5 - 25°C and were shown in the presence of 10 mM KNO3 (+N).

**Figure S3: Principal component analyses of gene expression profiles over time in (a) Bur and (b) Cvi.**  Genes are identified numerically as denoted; 1 = *ABI2*, 2 = *ABI3*, 3 = *ABI4*, 4 = *ABI5*, 5 = *CYP707A2*, 6 = *DOG1*, 7 = *FLC*, 8 = *Ga2ox2*, 9 = *Ga3ox1,* 10 = *GID1A*, 11 = *MFT*, 12 = *NCED6*, 13 = *NR1*, 14 = *NRT1*.1, 15 = *PIL5*, 16 = *PYL7*, 17 = *PYR1*, 18 = *RGA2*, 19 = *RGL2*, 20 = *Snrk2.1*, 21 = *Snrk2.4*, 22 = *SPT*, 23 = *CIPK23*, 24 = *PHYA*.
